# Disrupting *shadow* in the prothoracic gland induced larval development arrest in the fall armyworm *Spodoptera frugiperda*

**DOI:** 10.3389/fphys.2024.1502753

**Published:** 2024-12-11

**Authors:** Mian-Zhi Wu, Shu-Ting Fan, Yuan-Chen Zhang, Jin-Fang Tan, Guan-Heng Zhu

**Affiliations:** ^1^ School of Agriculture and Biotechnology, Shenzhen Campus of Sun Yat-sen University, Shenzhen, China; ^2^ State Key Laboratory of Biocontrol, School of Agriculture and Biotechnology, Sun Yat-sen University, Shenzhen, China; ^3^ College of Biology and Food Engineering, Anyang Institute of Technology, Anyang, China

**Keywords:** 20-hydroxyecdysone, critical weight, prothoracic gland, *shadow*, metamorphosis

## Abstract

**Introduction:**

The juvenile hormone (JH) and 20-hydroxyecdysone (20E) are the central regulating hormones of insect development. The timing of their secretion usually leads to developmental transitions.

**Methods:**

The developmental transitions were evaluated via the starvation treatment and the expressions of two key metamorphosis inducing factor in *Spodoptera frugiperda*. Then, the main endocrine organs, including the brain–corpora cardiacum–corpora allatum and prothoracic gland, were sampled from L4-24 h and L6-24 h larvae for the RNA-seq analysis. Additionally, the critical rate-limiting enzyme of 20E synthesis, *shadow*, was knocked down to mimic the downregulation of 20E synthesis in the late larval instar.

**Results:**

The critical weight (CW), when JH titer declines for metamorphosis, was determined be approximately L6-24 h in *S. frugiperda*. However, the expression of the pupal specifier *Broad-Complex* and the potential “metamorphosis initiation factor” *Myoglianin* showed a stepwise increase between L4-24 h and L6-24 h, suggesting that the developmental transitions may occur earlier. The RNA-seq analysis revealed that both 20E and JH synthesis enzymes were downregulated at the CW. In addition, strong tendencies in the expression pattern were detected among the lists of transcripts. Further knockdown of shadow induced larval development arrest and subsequent mortality, indicating that disrupting 20E synthesis before the CW is lethal. Besides, JH synthesis enzyme was down-regulated.

**Conclusion:**

The downregulation of 20E synthesis enzymes at the CW may represent a carefully regulated event, suggesting a deceleration of larval growth and the initiation of some underlying physiological changes to set the stage for metamorphosis.

## 1 Introduction

Insect molting and metamorphosis are regulated by two key hormones, namely, 20-hydroxyecdysone (20E) and the juvenile hormone (JH). Typically, an increase in 20E levels in the absence of JH triggers metamorphosis once the larvae reach the threshold size in the last instar (Truman and Riddiford, 2019). In contrast, in the presence of JH, the molting process leads to the production of another immature larval instar (Jindra et al., 2013). Although the threshold size required to initiate metamorphosis usually varies among species, most insects undergo similar size assessment events during their development (Mirth and Riddiford, 2007). One of the most well-defined of these events is the attainment of critical weight (CW), which marks the transition from growth to metamorphosis. The CW is defined as the minimal size of the larvae in the last instar, beyond which further feeding and nutritional intake are no longer required for a normal time course to initiate metamorphosis, and it is operationally determined by measuring the time it takes for the larvae subjected to starvation to enter the wandering stage (Nijhout and Callier, 2015). Larvae that are starved before reaching the CW can also manage delayed metamorphosis, using the extended time to allow the growth of the endocrine organs, such as the prothoracic glands (PGs), which produce sufficient ecdysone to halt growth and trigger metamorphosis (Mirth et al., 2005). Notably, the CW is not a result of simple nutrition accumulation for surviving metamorphosis but rather represents a developmental–physiological decision to switch the neuroendocrine activities. This shift includes a decrease in JH levels, an increase in JH esterase activity, and the secretion of ecdysone (Mirth and Riddiford, 2007). The elimination of JH and its residual effects require time, after which the release of the prothoracicotropic hormone (PTTH) and ecdysone synthesis are no longer inhibited. This is coupled with the cessation of feeding and the onset of wandering behavior (Nijhout and Callier, 2015). In earlier larval instars, the JH titer remains high and does not play a role in controlling the timing of PTTH and ecdysone secretion until another threshold size is reached. This threshold size occurs at the third (L3) or fourth (L4) larval instar in species such as *Bombyx mori*, *Aedes aegypti,* and *Tribolium castaneum*. When genetic manipulation or chemical allatectomy is used to create JH deficiency in these species, larvae undergo precocious metamorphosis, and the pupal specifier gene *Broad-Complex* (*BR-C*) is activated, committing the pupation process (Daimon et al., 2015; Konopova and Jindra, 2007; Zhu et al., 2019). Recently, this threshold size assessment event was identified in *Manduca sexta*, where larvae monitored their skeletal muscles as a proxy for body size. Once a critical muscle mass is reached, muscle tissues begin to release and accumulate *Myoglianin* (*Myo*) and eventually trigger the cessation of the growth by shutting down JH production at the CW (He et al., 2020). Whether or not *Myo* is related to the “metamorphosis initiation factor” that commits the larvae to metamorphosis remains unclear (Chafino et al., 2019; Martín et al., 2021; Smykal et al., 2014). However, it is hinted that these size assessment events are vital for uncovering the mechanisms by which the endocrine system prepares larvae for metamorphosis.

The main endocrine system controlling growth and metamorphosis includes the brain, corpora allatum (CA), and PG. The brain plays a central role in orchestrating signals from the body and releasing developmental instructions, such as PTTH (Truman and Riddiford, 2023). The CA, a pair of glands regulated by the brain, secretes JH at a certain time (Jindra et al., 2013). The PG, stimulated by PTTH, secretes the relatively inactive prohormone ecdysone (formerly called α-ecdysone), which is converted into the active hormone 20E (formerly β-ecdysone) in peripheral tissues like the fat body (Pan et al., 2021). It has been reported that the proper growth and maturation of both the brain and PG are indispensable for the normal initiation of metamorphosis (Cui et al., 2021; Mirth et al., 2005). However, the physiological changes and the underlying molecular events that lead to metamorphosis remain poorly understood. Transcriptional analysis of these organs may provide valuable insights, but the transcriptome data for these organs during the larva–pupa metamorphosis are limited. The few existing reports focus primarily on the specific developmental stage in *B. mori*, *Drosophila melanogaster*, *Helicoverpa armigera,* and *Antheraea pernyi* (Bian et al., 2022; Bian et al., 2019; Gao et al., 2022; Moulos et al., 2018). To address this gap, RNA-seq of the brain–corpora cardiacum–corpora allatum (BCC) and PG from larvae was conducted to identify additional biological processes or genes associated with metamorphosis in the study.

In this study, *Spodoptera frugiperda* was used to investigate the physiological changes in the late larval stages before metamorphosis. First, the CW of *S. frugiperda* larvae was determined through a starvation treatment in the late instars, identifying the developmental–physiological switch point for metamorphosis. Second, the developmental expression patterns of the pupal specifier gene *BR-C* and the potential “metamorphosis initiation factor” *Myo* were analyzed to figure out the duration of time when the initial endocrine changes lead to metamorphosis. Third, transcriptional analysis of BCC and PG in L4-24 h and L6-24 h was conducted to obtain more information about the endocrine changes at the CW. It was found that 20E synthesis and JH synthesis were reduced as larvae approached the CW. However, several physiological events, including the inhibition of ligand–receptor activity in the BCC, the inhibition of proteasome activity in the PG, and the activation of hydrolase and peptidase activity in the BCC, had already been initiated. As the critical rate-limiting enzyme of ecdysone synthesis, *shadow* was knocked down in L4 to mimic the downregulation of the 20E synthesis enzymes in the late larval instars. It resulted in significant larval development arrest and the simultaneous inhibition of JH synthesis. The results in the study described the developmental and physiological changes that occur as the last metamorphic molt approaches, and the downregulation of 20E synthesis enzymes at the CW suggested a deceleration in larval growth, initiating underlying physiological events that might be necessary for coordinating the timing of metamorphosis.

## 2 Materials and methods

### 2.1 Insect culture

*S. frugiperda* larvae were reared on an artificial diet (Pinto et al., 2019) at 26°C ± 1°C with a 60% ± 5% relative humidity and a photoperiod of 14-h light:10-h dark. To avoid cannibalistic behavior (He et al., 2022), the larvae were separated and reared individually in plastic containers after molting into the L3 instar. Larval molting was monitored every 4 h to accurately stage the larvae, and the newly molted larvae were traced and selected for the experiment at specific developmental stages. The formed pupae were sexed and kept in the plastic containers until eclosion. After eclosion, the moths were fed a 10% honey solution for mating and oviposition.

### 2.2 Starvation treatment

To perform starvation treatment, five developmental stages were selected, namely, L5-48 h, L6-0 h, L6-12 h, L6-24 h, and L6-48 h, to determine the entrance time of metamorphosis and the CW. At each designated stage, approximately 15 larvae per group were weighed and then transferred to the new plastic containers containing a 2% agar–water medium to induce starvation. For the control group, larvae at the same developmental stage were fed the artificial diet. All experiment larvae were traced and weighed daily until pupation or death. The final images of the larvae in each group were captured using a Nikon stereo microscope (SMZ745T).

### 2.3 RNA isolation

RNA isolation was performed with the TRIzol reagent (Thermo Fisher Scientific, Waltham, United States), according to the manufacturer’s instructions. The integrity and concentration of the RNA extracts were assessed using a NanoDrop 2000 spectrophotometer (Thermo Scientific, Wilmington, DE, United States) and an Agilent 2100 Bioanalyzer (Agilent Technologies, Santa Clara, CA, United States). After the premise of guaranteed RNA quality, an equal quantity of 1.0 μg of total RNA from each sample was reverse-transcribed into cDNA using the HiScript III 1^st^ Strand cDNA Synthesis Kit (+gDNA wiper) (Vazyme, China) for subsequent quantitative real-time polymerase chain reaction (qRT-PCR).

### 2.4 Quantitative real-time polymerase chain reaction

qRT-PCR was performed using the AceQ Universal SYBR qPCR Master Mix (Vazyme, China) with three technical replicates on the QuantStudio 5 Real-Time PCR System (Thermo Fisher Scientific, Waltham, United States). Gene-specific primers used for qRT-PCR analysis are listed in Supplementary Table S1. The relative mRNA expression levels of the target genes were calculated with the 2^−ΔΔCT^ method (Livak and Schmittgen, 2001) and normalized to the expression of the housekeeping genes *RPL-10* and *α-tubulin* (Han et al., 2021).

### 2.5 Morphological observation

The PG and BCC were dissected from larvae at L4-24 h, L5-24 h, and L6-24 h. After washing with cold phosphate-buffered saline (PBS) (Coolaber, China), the tissues were fixed in 4% paraformaldehyde at 4°C for 30 min. The fixed tissues were then mounted on the microscope slide and stained with DAPI (Servicebio, China) for 1 h. The slides were visualized on an Olympus stereo microscope (SZX16-DP74) to examine the morphological changes.

### 2.6 Preparation and sequencing of mRNA libraries

To perform the RNA sequencing of PG and BCC, 15 larvae at specific developmental stages were selected as one biological replicate, and three biological replicates of each group were prepared. After the RNA isolation of the collected samples, the integrity of the RNA extracts was assessed, and the samples that met quality standards were used to construct the cDNA libraries for transcriptome sequencing analysis (Frasergen, Wuhan, China). In brief, poly(A)+ mRNA, enriched by immobilized Oligo dT, was fragmented and reverse-transcribed into double-stranded cDNA. The ends of these cDNA were blunted, and an overhang “T” was added at the 3′ end. The products were then denatured to form single-stranded DNA and looped with a bridge primer to generate the single-stranded circular DNA libraries.

After the construction of the DNA libraries, the quality was evaluated by measuring concentrations and size distributions. The concentrations were measured by qPCR, and the size distribution of the libraries was analyzed using the Agilent 2100 Bioanalyzer system (Agilent, United States). Once the library passed quality control, they were loaded onto a sequencing chip and sequenced using an MGI high-throughput sequencer. Subsequently, adapters, ambiguous nucleotides, and low-quality sequences were removed, and the obtained clean reads were aligned with the reference genome of *S. frugiperda* (GenBank ID: GCA_023101765.3). HISAT2 software was used to align the spliced reads with sequences in the reference genome, achieving a total mapped ratio of over 82% for the BCC and PG transcriptomes, indicating that the spliced reads belonged to *S. frugiperda*. In addition, multiple mapped and discordant reads were screened from the raw data and removed, and the remaining alignments were examined for the significance analysis of differential gene expression using DESeq2 software.

### 2.7 RNA interference

Small interference RNAs (siRNAs) efficiently induce gene silencing in other lepidopterans (Fu et al., 2022). The siRNA sequences targeting *shadow* were designed using siDirect 2.1 (https://sidirect2.rnai.jp/) based on the verified sequences (accession number: LOC118269113; sequences listed in Supplementary Table S1). These siRNAs were chemically synthesized by Shanghai GenePharma Co., Ltd. (Shanghai, China) and dissolved in diethyl pyrocarbonate-treated water (Milli-Q grade) to a concentration of 4 μg/μL. Forty larvae were randomly selected for each group, with a total of 80 larvae used in the experiment. When the larvae molted into L4-0 h, a volume of 1 μL of the siRNA solution was injected into the ventral part of the intersegment membrane between the first and second thoracic segments using a micro-syringe. The negative control (NC)-siRNA was used as a control. The injection was repeated 24 h later on the same individuals to enhance the effectiveness of the treatment. The treated larvae were maintained separately in the laboratory under standard conditions and observed daily for potential phenotypic changes induced by target gene silencing. Meanwhile, 15 PGs in L4-44 h RNAi larvae and 10 PGs in L4-68 h RNAi larvae were dissected to assess the RNAi efficiencies at specific developmental stages.

## 3 Results

### 3.1 Only starved larvae in the last instar can survive to molt into pupae

**FIGURE 1:**
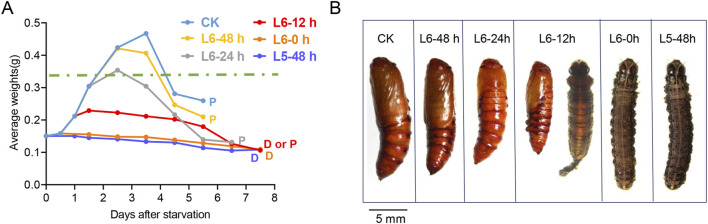
Determination of the critical weight in the L5 and L6 instar of *S. frugiperda*. **(A)** Growth curves of larvae in the starvation treatment. Groups of larvae were starved at different developmental stages in the late instars, and their daily average weights were recorded and are shown with lines of different colors. An average of 15 larvae were manipulated in each group of the treatment. The data were tracked until the larvae died (marked with “D”) or pupated (marked with “P”). “D or P” indicates that both cases occur in the same group. The predicted critical weight is represented with a green dashed line. **(B)** Representative images of the forming pupae or dead larvae in the starvation treatment. The stages are shown at the top of each image, and the images indicate the pupation of each group in the experiment. A pupa from the feeding larva is also exhibited on the left for control (scale bar: 5.0 mm).

During the late larval instars, the CW is a crucial size assessment event that determines whether a larva is ready to progress to the next developmental stage or requires further growth (Mirth and Riddiford, 2007). However, the CW of *S. frugiperda* has not been previously measured. To establish a precise weight range for the CW, starvation treatment was carried out to mimic the size assessment process of *S. frugiperda*. Hatching larvae were fed the artificial diet until they developed into the specific stages selected for the treatment. Subsequently, these larvae were provided with a water diet consisting of 2% agar until they either began the metamorphic molt or succumbed to starvation. The water diet was used as a supply of water to keep the larvae hydrated and prevent death from thirst. Generally, larvae in the laboratory cease growth and enter a wandering phase between 48 and 72 h after molting into the L6 instar, approximately 60 h before pupation (Figure 1A). During starvation treatment, the larvae could hardly initiate metamorphosis or form pupae until they developed into L6-12 h. However, most of the experiment larvae at L6-12 h failed to complete pupation and died with a delay of 1–2 days (Figure1 A). Notably, half of the larvae at L6-24 h (8/16) managed a normal time course to metamorphosis, albeit at a smaller size (Figure1 B). Almost all larvae at L6-48 h (14/15), after an additional 24 h of feeding, initiated metamorphosis at normal times, suggesting that they had attained the post-critical weight (Figure1 A). Thus, the threshold size, leading to a timely metamorphic molt, appears to be ranged amongst the larvae at L6-24 h. It is worth noting that the weight loss of the larvae occurred after 1–2 days of starvation (Supplementary Table S2), which may depend on the amount of food remaining in their guts. Therefore, the last measured weight before the onset of weight loss of the starved larvae was selected to estimate the CW. The precise weight range for the CW is estimated to be between 0.348 g and 0.367 g.

### 3.2 20E synthesis and JH synthesis were not activated at the time of the CW

The CW represents a developmental “check-point” that, once reached, triggers an endocrine cascade leading to the cessation of feeding and the initiation of metamorphosis (Nijhout and Callier, 2015). It has been reported that this metamorphic checkpoint is associated with a decrease in JH titers, driven both by the inhibition of JH biosynthesis and the emergence of JH-specific esterases in the hemolymph (Mirth and Riddiford, 2007; Nijhout and Callier, 2015). However, larvae experiencing delayed metamorphosis did not immediately cease their growth but instead showed a gradual decrease in weight before finally undergoing metamorphosis. This suggests a degree of flexibility in the endocrine system during this transition.

**FIGURE 2:**
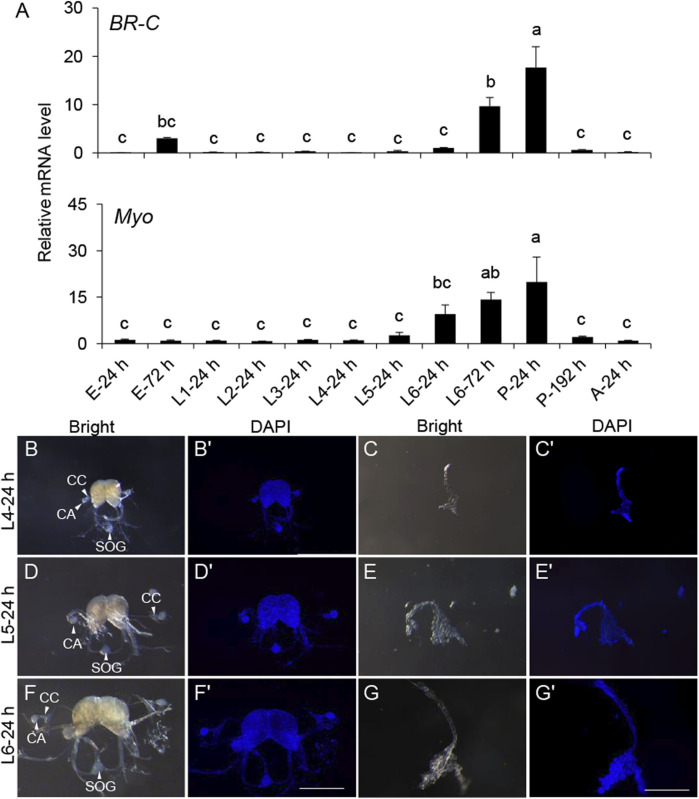
Gene expression patterns of *BR-C* and *Myo* in the development and morphology of the endocrine organs BCC and PG in the late instar of *S. frugiperda*. **(A)** Expression levels of *BR-C* and *Myo* mRNA in diverse developmental stages. The stages of the samples are shown below. The results represent the mean ± SE values (n = 3 biological replicates). “E,” embryonic stage; “L,” larval stage; “P,” pupal stage; “A,” adult stage. The result of L4-24 h was used as a calibrator in the 2^−ΔΔCt^ quantification method. One-way ANOVA Tukey’s *post hoc* test was used to assess the significance of the changes in mRNA levels. Bars with the same letters are not significantly different at 95% CI. **(B,D,F)** Morphology of the BCC from L4, L5, and L6 instar larvae (scale bar: 0.5 mm). The corpora allatum (marked with “CA”), corpora cardiacum (marked with “CC”), and sub-esophageal ganglion (marked with “SOG”) are indicated by white solid arrows or marked directly in the images. **(C,E,G)** Morphology of the PG from L4, L5, and L6 instar larvae (scale bar: 0.5 mm). **(B′–G′)** The corresponding morphology of the BCC or PG was stained by DAPI (scale bar: 0.5 mm). “Bright,” organs photographed in the bright field. “DAPI,” organs stained with DAPI.

To further explore this, a developmental gene expression profile was carried out. The expression of the pupal specifier gene, *BR-C*, was transiently detected before the L6 instar and significantly increased from L6-24 h. *BR-C* expression peaked at the onset of pupation, coinciding with the pupal development (Figure 2A), indicating its conserved role in *S. frugiperda*. *Myo* maintained a basal expression level before L4-24 h but sharply increased at L6-24 h and reached the top until the completion of pupation (Figure 2B). This developmental gene expression profile implied that the period between L4-24 h and L6-24 h might be associated with some expected physiological changes necessary for the initiation of metamorphosis.

Morphology of the main endocrine organs was also identified to seek out more clues at this period. The major components of the neurosecretory system—BCC and PG—were dissected from the L4, L5, and L6 instar larvae for morphological characterization. These organs showed a significant increase in size as development progressed through the late larval instars. However, aside from the increase in size, no notable morphological changes were observed (Figures 2C–G).

**FIGURE 3:**
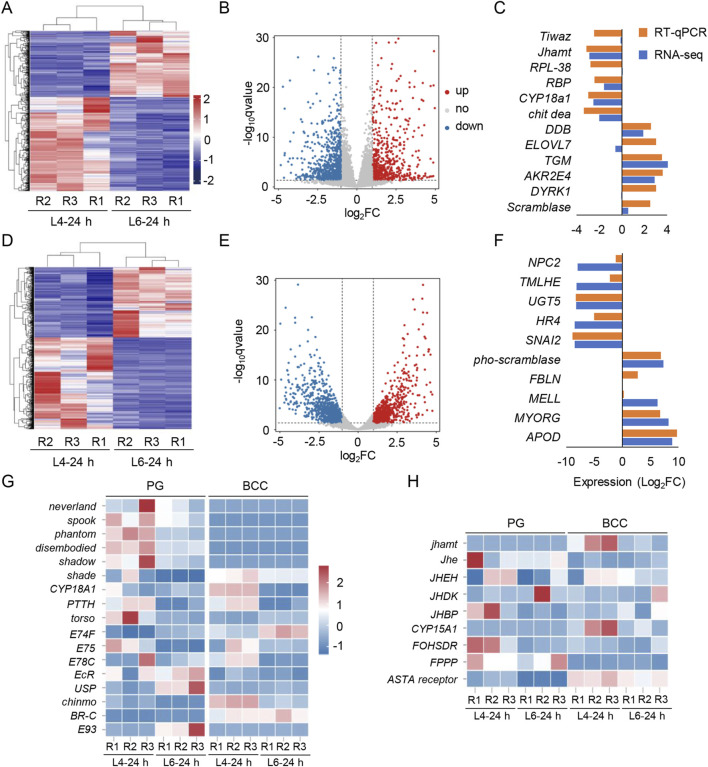
Transcriptome screening for the BCC and PG and the transcripts of the 20E and JH pathway in *S. frugiperda*. **(A,D)** Heatmaps display the expression levels of mRNAs in the BCC and PG from L4-24 h and L6-24 h. R1–R3 represent three biological replicates, and the clustering analysis is presented in the maps. **(B,E)** Volcano plots of DEGs in two transcriptomes. The red and blue dots indicate the upregulated and downregulated transcripts, respectively [log_2_ (fold change) ≥ 1; *p* < 0.05]. **(C,F)** Genes selected in the BCC and PG transcriptomes were verified by qRT-PCR and compared with the results of the transcriptomes. The data are shown as the mean (RNA-seq, n = 3; RT-qPCR, n = 3). The gene name abbreviations are shown, and the ID of these genes is listed in Supplementary Material S2. **(G)** Heatmaps of transcripts characterized in the “20E signaling pathway” include the synthesis pathway of the ecdysone, PTTH, and 20E signaling pathway, ecdysone receptors, and stage-specifying master genes. **(H)** Heatmaps of transcripts characterized in the “JH signaling pathway” include the synthesis and metabolism pathway of JH and methyl farnesoate. R1−R3 represent three biological replicates. The name abbreviations of the transcripts are shown on the left side of the heatmaps, and the corresponding full names are listed in Supplementary Material.

To explore molecular changes associated with metamorphosis, RNA-seq of BCC and PG from larvae at L4-24 h and L6-24 h was further done. The raw data on RNA-seq were uploaded to the NCBI under the accession number PRJNA1173108. Using a *p*-value threshold of 0.05 (FDR or adjusted *p*-value) and an absolute log_2_ (fold change) value of 1.0, 2,453 and 2,622 differentially expressed genes (DEGs) in the BCC and PG were identified, respectively (Figures 3A, D). Among them, 1,167 DEGs in the BCC and 1,351 DEGs in the PG were upregulated in the L6-24 h *versus* L4-24 h group, while 1,286 DEGs in the BCC and 1,271 DEGs in the PG were downregulated (Figures 3B, E).

To confirm the accuracy of the RNA-seq findings, 22 transcripts were randomly selected from each transcriptome for qRT-PCR analysis. Out of the 12 genes selected from the BCC transcriptomes and 10 from the PG transcriptomes, expression measurements were successfully obtained. The results of qRT-PCR were consistent with those of RNA-seq for seven genes in the BCC transcriptome and six genes in the PG transcriptome, confirming the reliability of the RNA-seq analysis (Figures 3C, F).

JH and 20E are the two key hormones that coordinate the metamorphic processes, acting antagonistically (Truman and Riddiford, 2019). To investigate the changes in these two hormones around the metamorphic phase, transcripts of 20E pathways were first identified in the transcriptomes, including those with insignificant changes (Figure 3D). The Halloween genes, which are involved in 20E biosynthesis—s*pook*, *phantom*, *disembodied*, and *shadow*—were downregulated in L6-24 h compared with L4-24 h in the PG transcriptomes. In addition, another Halloween gene, *shade*, which is expressed in the peripheral nervous system and involved in the conversion of ecdysone to its active form, 20E, was also downregulated in L6-24 h compared with L4-24 h in both transcriptomes.

The downward trends of these Halloween genes implied a reduction in 20E synthesis, which was consistent with the observed downregulation of the transcript of *EcR* and its downstream effectors, *ecdysone-inducible protein 75* (*E75*) and *ecdysone-induced protein 78C* (*E78C*), in L6-24 h PG transcriptomes. However, the transcript levels of the second partner of the ecdysone receptor, *Ultraspiracle* (*USP*), and one of its downstream effectors, *ecdysone-induced protein 74* (*E74*), were upregulated in the PG transcriptomes. Interestingly, the expression of *cytochrome P450 protein 18A1* (*CYP18A1*), a key cytochrome P450 enzyme involved in the inactivation of 20E (Guittard et al., 2011), was sharply reduced in the BCC transcriptomes upon developing into L6-24 h. Additionally, *PTTH* and its receptor *torso*, which stimulated PG glands to produce ecdysone, were detected as insignificant in the BCC transcriptomes, further supporting the inactivation of 20E synthesis at this stage.

Transcripts of JH pathways were also identified in the transcriptomes (Figure 3H). As the critical rate-limiting enzyme for JH biosynthesis, *JH acid o-methyltransferase* (*jhamt*) controls JH levels in most insects. It was only detected in the BCC transcriptomes and downregulated in L6-24 h compared with L4-24 h in the BCC transcriptomes. Three enzymes that generate the JH direct precursor methyl farnesoate, namely, *cytochrome P450 protein 15A1* (*CYP15A1*), *farnesol dehydrogenase* (*FOHSDR*), and *farnesyl diphosphate phosphatase* (*FPPP*), showed a lower expression level in the BCC transcriptomes at L6-24 h, implying that JH biosynthesis was not activated at the time. Meanwhile, the transcripts of *JH esterase* (*Jhe*) and *JH epoxide hydrolase* (*JHEH*), two enzymes that efficiently degrade JH, were barely detected in either transcriptome, which hinted that JH clearance in these organs had not been initiated yet.

### 3.3 Underlying endocrine events that might contribute to the metamorphic initiation

The downward trends of 20E and JH clearance transcripts in L6-24 h compared with L4-24 h might provide insights into the physiological status in the late larval instars. To seek more details of the status, functional annotation was performed in both transcriptomes, and some enriched DEGs in the transcriptomes could be clustered in a few Gene Ontology (GO) terms, which showed a strong tendency in the expression pattern.

**FIGURE 4:**
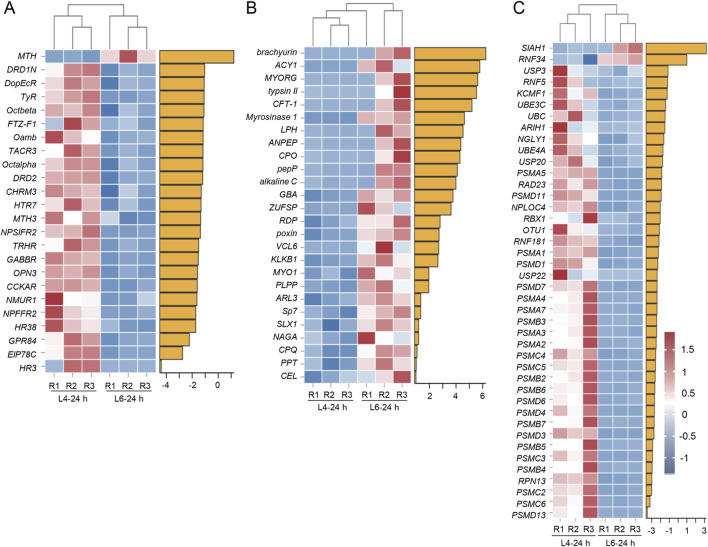
Heatmaps of representative transcripts in BCC and PG transcriptomes in L4-24 h and L6-24 h in *S. frugiperda*. **(A)** Heatmaps of representative transcripts identified in the “Ligand–receptor activity” term from the BCC transcriptome. **(B)** Heatmaps of representative transcripts identified in the “Hydrolase and peptidase activity” term from the BCC transcriptome. **(C)** Heatmaps of representative transcripts identified in the “Proteasome activity” term from the PG transcriptome. R1−R3 represent three biological replicates. The name abbreviations of the transcripts are shown on the left side of the heatmaps, and the corresponding full names are listed in Supplementary Material. The yellow horizontal bars represent the log_2_FC value of the corresponding gene.

In the BCC transcriptomes, almost all DEGs identified in the GO term “Ligand–receptor activity” were downregulated in L6-24 h compared to L4-24 h in the BCC transcriptomes. This included receptors such as dopamine receptors (*DRD1N* and *DRD2*), octopamine receptors (*Octalpha* and *Octbeta*), thyrotropin-releasing hormone receptors (*TRHR*), neuropeptide receptors (*NPSIFR2*), and G-protein-coupled receptors (*MTH4*, *DopEcR, MTH3*, and *GPR84*). In addition, members of the nuclear receptor superfamily regulated by 20E, such as *fushi tarazu factor-1* (*FTZ-F1*), *hormone receptor 3* (*HR3*), and *hormone receptor 38* (*HR38*), were also identified in this term (Figure 4A). “Hydrolase and peptidase activity” term, on the other hand, enriched all upregulated DEGs in the BCC transcriptomes, comparing L6-24 h and L4-24 h, and some of them are peptidases, like *aminopeptidase* (*ANPEP*), *zinc carboxypeptidase* (*CPO*), *pro-aminopeptidase* (*pepP*), and *carboxypeptidase Q* (*CPQ*). Others like *trypsin II*, *chicken fucosyltransferase 1* (*CFT-1*), *alkaline C*, *kallikrein 1* (*KLKB1*), and *serine protease 7* (*Sp7*), are serine-type endopeptidases participating in protein degradation (Figure 4B).

In the PG transcriptomes, there was a cluster of DEGs identified in “proteasome activity” that were downregulated in L6-24 h compared with L4-24 h. Most of them were 26S proteasome subunits, and several were E3 ubiquitin–protein ligases, including *SIAH1*, *ring finger protein 34* (*RNF34*), *ring finger protein 5* (*RNF5*), *potassium channel modulatory factor 1* (*KCMF1*), *ariadne-1* (*ARIH1*), *ring box protein* (*RBX1*), and *ring finger protein 81* (*RNF181*) (Figure 4C).

### 3.4 Inhibition of 20E synthesis disturbed JH signaling and arrested the larval development

**FIGURE 5:**
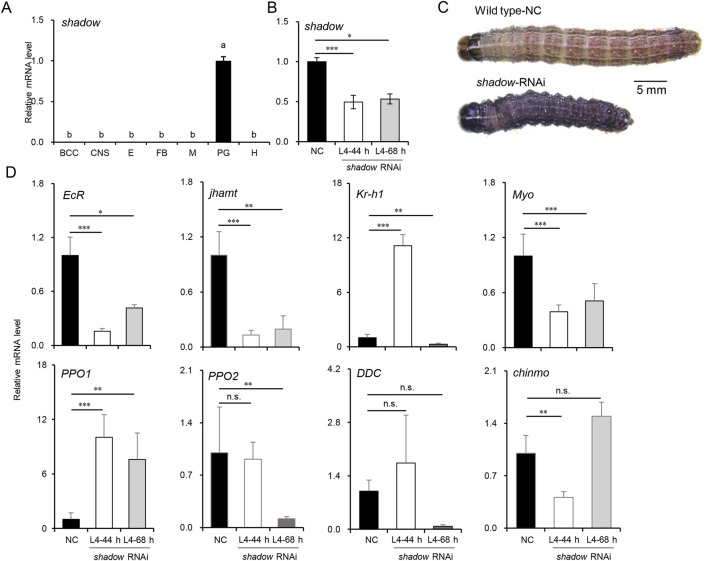
Knockdown of *shadow* disturbed JH signaling and delayed the larval development in L4 instar larvae of *S. frugiperda*. **(A)** Expression of *shadow* in tissues of L4-24 h larvae. “BCC,” brain, corpora cardiac, and corpora allatum; “CNS,” central nervous system; “E,” epidermis; “FB,” fat body; “M,” Malpighian tubule; “PG,” prothoracic gland; “H,” hemolymph. One-way ANOVA Tukey’s *post hoc* test was used to assess the significance of the changes, as in Figure 2A. **(B)** Expression of shadow at 44 h and 68 h after the first injection of siRNAs. “***” denotes the significant differences of mRNA levels between control and *shadow* RNAi at *p* < 0.001 analyzed by using Student’s *t*-test. Mean ± SE (n = 3) is shown. **(C)** Phenotypes of *shadow*-depleted larvae in L4 (scale bar: 5 mm). **(D)** Expression patterns of genes associated with development and melanization in L4-44 h and L4-68 h larvae after *shadow* RNAi. Mean ± SE (n = 3) is shown, and “*,” “**,” and “***” denote the significant differences in mRNA levels between control and *shadow* RNAi at *p* < 0.05, *p* < 0.01, and *p* < 0.001 analyzed by using Student’s *t*-test, respectively.

Most enzymes involved in 20E and JH syntheses decreased as larvae developed into the CW, suggesting an endocrine switch linked to metamorphosis. The inhibition of *JHAMT* or other biosynthetic enzymes in the late larval stages induces precocious metamorphosis in *B. mori* (Daimon et al., 2012; Daimon et al., 2015), and the phenotypes resulting from disrupted JH signaling by the knockdown of *Met* or *Kr-h1* have been well-described in the larvae of *B. mori and T*. *castaneum* (Daimon et al., 2015; Konopova and Jindra, 2007), reinforcing the idea that JH biosynthesis is required to maintain the normal larval development in the late larval stages. However, the molecular mechanism of 20E synthesis during this stage has not been fully discussed. It has been proven that the expression of *disembodied* and *shadow* was restricted to PG as the critical rate-limiting enzymes of 20E synthesis (Gilbert, 2011; Pan et al., 2021). Moreover, *shadow* could be efficiently knocked down in lepidopterans (Peng et al., 2019). To better understand the biological process, siRNAs were used to knock down the *shadow* gene, which was highly and specifically expressed in PG (Figure 5A). The siRNAs were injected into newly molted L4-0 h larvae to suppress *shadow* expression, and a second injection was performed after 24 h to enhance the knockdown effect. Typically, the L4 instar lasts 48–56 h under laboratory conditions, and control larvae molted into L5 at approximately 50 h in the experiment. Thus, L4-44 h was chosen for sampling. The PGs from 15 injected larvae were dissected for total RNA isolation at this time; then, the knockdown efficiencies were confirmed *via* qRT-PCR. As shown in Figure 5B, the knockdown efficiency exceeded 50%, significantly inhibiting the growth of the injected larvae. Twelve out of 15 *shadow* RNAi larvae extended their growth in L4 for an additional 24–72 h compared to the control larvae. Ten RNAi larvae at L4-68 h were subsequently sampled. Interestingly, compared to the control larvae at L4-44 h, many of the *shadow* RNAi larvae at both L4-44 h and L4-68 h displayed a melanized phenotype (9/15), potentially due to the interactions between the hormone and melanin pathway, and most RNAi larvae experienced developmental arrest after the injection (Figure 5C). Finally, fewer than 20% of injected larvae (2/15) survived to molt into the L5 instar.

The differential expression patterns of genes associated with development were also analyzed to assess the impact of *shadow* depletion (Figure 5D). *EcR* expression decreased sharply and stayed below 50% of its control level, implying that the 20E signal was significantly reduced after the silencing of *shadow*. The expression level of *jhamt* was also affected by the *shadow* RNAi and decreased to 20% of its control level. Interestingly, the transcript of the JH-inducible gene, *krüppel-homolog 1* (*Kr-h1*), was temporarily activated in L4-44 h but returned to a lower level 24 h after the knockdown of the *shadow* gene. The potential “metamorphosis initiation factor” *Myo* was inhibited and maintained at a low level after *shadow* knockdown. To better understand the molecular mechanisms behind the melanized phenotype observed in the *shadow* RNAi larvae, the expression levels of *dopa decarboxylase* (*DDC)*, *pro-phenoloxidase 1* (*PPO1),* and *pro-phenoloxidase 2* (*PPO2*) were measured. Remarkably, after knocking down the *shadow* gene, the expression of the *PPO1* gene increased approximately 10-fold. At the same time, *PPO2* was downregulated after knocking down the *shadow* gene. Interestingly, the expression of the *DDC* gene fluctuated in the control larvae, suggesting potential instability in the melanin production pathway. Previous studies in *Ae. aegypti* found that JH suppression does not induce the melanized phenotype in the late larval stages (Zhu et al., 2019). In this study, after knocking down the *shadow* gene, the differentially expressed *jhamt* and *Kr-h1* genes indicated that the antagonistic regulation between JH and 20E is out of control (42). This dysregulation contributed to the upregulation of the *PPO1* gene, resulting in a melanized larval phenotype. Additionally, the newly reported larval master gene, *chronologically inappropriate morphogenesis* (*chinmo*) (Reynolds, 2022), was temporarily inhibited after *shadow* RNAi, which might support the slow growth of the *shadow* RNAi larvae.

## 4 Discussion

Insect metamorphosis is orchestrated by JH and 20E and is triggered when larvae reach a specific threshold size called the CW. In this study, the CW of *S. frugiperda* was first measured and found to be between 0.348 g and 0.367 g, approximately at L6-24 h. Larvae that were starved and failed to reach the CW were unable to initiate metamorphosis on time, and those starved earlier than L6-12 h would never enter the wandering stage. The larvae between L6-12 h and L6-24 h, which experienced delayed metamorphosis, likely surpassed the critical period for sufficient nutrition in the development (Mirth and Riddiford, 2007); however, their organs, particularly the PG and brain, were underdeveloped (Cui et al., 2021; Mirth et al., 2005). Therefore, it caused a delay in entering the wandering stage until they reached maturity. In fact, there is another concept called minimal viable weight (MVW), which represents the minimal weight at which larvae have sufficient fat body storage to survive metamorphosis. The MVW usually takes place before the CW, and the larvae that are starved after reaching the MVW but before the CW will experience a prolonged and fail-prone metamorphosis. In the study, the starved larvae in the L6-12 h group and some in the L6-24 h group, which did not surpass the CW, showed delayed metamorphosis, and most of them died in the form of a larva/pupa intermediate. This suggests that they might reach the MVW but fail to surpass the CW. However, the exact range of the MVW in *S. frugiperda* could not be determined due to limited data. Even in well-studied species like *D. melanogaster*, which can be fed under well-established conditions, and the life stages of which can be precisely timed in an hour, the MVW has not been determined to date. The MVW is believed to be a developmental stage rather than a specific time point in the last larval instar, and it is highly dependent on the nutritional storages of the individual.

Although CW has been studied for decades, it has only been confirmed in a few species like *M. sexta* and *D. melanogaster*. In all cases, larvae that surpass CW are able to manage a normal time course to metamorphosis (Callier and Nijhout, 2013; Mirth et al., 2005; Nijhout and Williams, 1974). The physiological processes leading to the secretion of ecdysone and the cessation of growth begin at the CW, and it usually takes time for JH synthesis to be suppressed and the JH titer to clear (Nijhout and Callier, 2015). To explore these processes and confirm the duration of the switch, a developmental expression pattern and morphological changes were performed. The induction of *BR-C* and *Myo* occurred around L4-24 h and L6-24 h in this study, indicating some underlying physiological events might take place within this developmental window.

A further RNA-seq analysis of the main endocrine organs, including BCC and PG, was manipulated at L4-24 h and L6-24 h. Unexpected downregulation of 20E synthesis and JH synthesis enzymes were detected in the BCC and PG transcriptomes in L6-24 h compared with L4-24 h. Critical rate-limiting enzymes for JH synthesis and 20E synthesis, *JHAMT* and *shadow*, were particularly notable for their downregulation. In addition, DEGs associated with several potential physiological events, like the inhibition of ligand–receptor activity, the activation of hydrolase and peptidase activity in the BCC, and the inhibition of proteasome activity in the PG, were identified. They exhibited strong tendencies in the expression pattern. Mirth and Riddiford (2007) reported that the reduction in JH titer occurs when larvae reach the CW. This reduction is due to the cessation of JH synthesis by the CA and the activation of JH esterase in the hemolymph. The downregulation of that and other *JH* synthesis enzymes in the BCC transcriptomes suggested that the anti-metamorphic effect of JH was not necessary at this time and implied a switch from larval growth to metamorphosis.

Similarly, the main enzymes involved in 20E synthesis in the PG, such as *spook*, *phantom*, *disembodied*, and *shadow,* were also downregulated in the L6-24 h transcriptomes compared to the earlier larval stage. Rather than the processes after the time when the anti-metamorphic effect of JH was dismissed, it was closer to the phenomena reported in *B. mori* and *M. sexta*, where JH synthesis gradually decreased and ceased at a certain time when the 20E titer became a trace level after the last larval ecdysis (Kaneko et al., 2011; Kinjoh et al., 2007; Nijhout, 1998). It is possible that the downregulation of 20E synthesis enzymes at the CW triggers a reduction in JH synthesis enzymes. The circulating JH levels, at this time, have not been cleared yet, so the elevation of 20E titers for metamorphosis would be activated later. Although the circulating 20E levels at CW have not been measured in this study, the observed downregulation of 20E synthesis enzymes in the PG transcriptomes might be linked to the concurrent downregulation of JH synthesis enzymes.

The tendentious expression pattern of DEGs in the transcriptomes hint at a physiological switch during the CW stage. The ligand–receptor-related genes identified in the BCC transcriptomes showed a significant inhibition in L6-24 h compared with L4-24 h. Most of these genes play crucial roles in development regulation (Granger et al., 1996), and they are inactivated when the 20E titer is low (Hiruma et al., 2013). Meanwhile, DEGs identified in the GO term “hydrolase and peptidase” were upregulated in the BCC transcriptomes. These genes code the enzymes that are important in peptidergic signaling systems in the brain, particularly for clearing neuropeptides, which are essential for modulating the neurotransmitter/modulator during development (Isaac et al., 2009). There may be a correlation between the decrease in the ligand–receptor activity and the increase in the hydrolase and peptidase activity, which implied an initiative deceleration in larval growth, although further investigation is necessary to confirm this hypothesis. However, the changes in these genes did imply that some underlying physiological events take place at the CW.

The PG is a crucial endocrine organ that produces ecdysone, a precursor to 20E, and integrates various signals that initiate molting and metamorphosis (Truman and Riddiford, 2019). The downregulation of genes identified in the GO term “proteasome activity” in L6-24 h compared with L4-24 h in the PG transcriptomes suggested that proteolysis may be suppressed at this time. Proteasome-mediated protein degradation is usually active during insect development and plays a crucial role in various physiological processes (Kanost and Clem, 2012). The inhibition of proteasome genes in L6-24 h could hint at a switch in the development as reduced proteolysis could be associated with the feedback inhibition of 20E synthesis, a phenomenon observed in *B. mori* larvae during the penultimate instar. This inhibition is thought to be shut down before the increase in the 20E titer in the last instar (Takaki and Sakurai, 2003). It could be assumed that the inhibition of the proteolysis genes at L6-24 h was some constituent part of such a feedback inhibition. The inactive proteolysis before the initiation of metamorphosis would be called back when some clearance events took place.

The silence of 20E synthesis in the late larval instars provides more clues. One of the rate-limiting enzymes of 20E synthesis, *shadow*, was knocked down in the L4 larvae. In the previous experiment, it usually took 24–72 h for good performance after siRNA injection. Thus, L6 larvae seemed too old for the experiment. Instead, L4 larvae were chosen in the experiment. Injecting s*hadow* siRNA into the L4 larvae delayed larval development, causing most larvae to fail to molt into the L5 instar. These phenotypes mirror those seen in other species when 20E synthesis is disrupted (Peng et al., 2019; Sugahara et al., 2017; Wan et al., 2015). Furthermore, the knockdown of *shadow* led to reduced JH synthesis, consistent with the results in the BCC transcriptomes in L6-24 h. This reduction in JH, along with the decreased expression of the potential “metamorphosis initiation factor” *Myo* and the larval master gene *chinmo*, supports the notion that the 20E synthesis disruption halts larval development. It is well-known that inhibiting JH synthesis before the last instar can induce precious metamorphosis (Daimon et al., 2012; Truman and Riddiford, 2019). However, when 20E synthesis was disrupted at the same time in this study, larval development was arrested, and death occurred in the experimental larvae before reaching the CW. It indicates that maintaining both 20E and JH syntheses was indispensable for normal larval development. The downregulation of 20E synthesis enzymes at the CW appears to be a carefully regulated event, signaling the end of the need for feeding and nutrition for timely metamorphosis. It might also hint at the deceleration of larval growth and induce some underlying physiological events in the BCC and PG to set the stage for metamorphosis.

## 5 Conclusion

Our study described the developmental and physiological events around metamorphosis in *S. frugiperda*. The CW of *S. frugiperda* was determined, which indicated a threshold size when feeding and nutrition are no longer required for a normal time course to reach metamorphosis and when growth would be set aside for other physiological processes of the metamorphosis. At this time, larval growth begins to decelerate, and while JH synthesis gradually decreased, 20E synthesis was not activated yet. Other potential events, like the inhibition of ligand–receptor activity in the BCC, the inhibition of proteasome activity in the PG, and the activation of hydrolase and peptidase activity in the BCC, were also identified in the transcriptomes. Disturbing *shadow* expression in the late larval instars arrested larval development and inhibited JH synthesis, which suggested that the downregulation of 20E synthesis enzymes at the CW was a carefully regulated event. It appears to be a pivotal event that signals a stepwise deceleration of the growth and initiates some physiological processes to set the stage for metamorphosis.

## Data Availability

The original contributions presented in the study are publicly available. The transcriptome data has been deposited on the NCBI repository, accession number is PRJNA1173108.
