# Supplementary material for: Disrupting shadow in the prothoracic gland induced larval development arrest in the fall armyworm Spodoptera frugiperda
Source: Front Physiol. 2024 Dec 11;15:1502753. doi: 10.3389/fphys.2024.1502753 (PMC11668756; doi:10.3389/fphys.2024.1502753)
Supplement: Supplementary file 1 [file Table1.docx]

*Supplemental data to*

**Disrupting *shadow* in the prothoracic gland** **induced larval development arrest in the fall armyworm, *Spodoptera frugiperda***

Mian-Zhi Wu^1,2^, Shu-Ting Fan^1,2^, Yuan-Chen Zhang^3^, Jin-Fang Tan^1,2^ and Guan-Heng Zhu^1,2 *^

*^1^ School of Agriculture and Biotechnology, Shenzhen Campus of Sun Yat-sen University, No. 66, Gongchang Rd, Guangming District, Shenzhen, 518107, China*

*^2^ State Key Laboratory of Biocontrol, School of Agriculture and Biotechnology, Sun Yat-sen University, Shenzhen 518107, China*

*^3^ College of Biology and Food Engineering, Anyang Institute of Technology, Anyang, China*

*Corresponding author: Guan-Heng Zhu E-mail: zhugh8@mail.sysu.edu.cn

**Table S1. The list of primers used in this study**

| **Primer** | **Sense (5'-3')** | **Antisense (5'-3')** | **Purpose** |
| --- | --- | --- | --- |
| *RPL-10*-qF | TGGGTAAGAAGAAGGCTACG | CGTAGCCTTCTTCTTACCCA | qRT-PCR for *BR-C* and *Myo* |
| *RPL-10*-qR | TGTTGATGCGGATGACAT | ATGTCATCCGCATCAACA |  |
| α-Tub-qF | AGGGCTGTGTTTGTTGACT | TCCCGACACAAACAACTGA |  |
| α-Tub-qR | TCCTTACCGATGGTGTAGTG | AGGAATGGCTACCACATCAC |  |
| *Myo*-qF | TAATTCCTTGCGTGTGGGCT | AGCCCACACGCAAGGAATTA |  |
| *Myo*-qR | GGCACAACCAAACTCATTC | GAATGAGTTTGGTTGTGCC |  |
| *BR-C*-qF | ACAAAGAACAACACCCACGC | GCGTGGGTGTTGTTCTTTGT |  |
| *BR-C*-qR | ATCGATGTCCGGGTTGGATG | CATCCAACCCGGACATCGAT |  |

**(A) Developmental gene expression profiles**

**(B) Validation of the BCC transcriptomes**

| **Primer** | **Sense (5'-3')** | **Antisense (5'-3')** | **Purpose** |
| --- | --- | --- | --- |
| *shadow*-F | CAGCACCCAAGTCTATTGAA | TTCAATAGACTTGGGTGCTG | Sequence verification |
| *shadow*-R | AATAAATTACTCACGCCTCC | TTCAATAGACTTGGGTGCTG |  |
| *shadow*-siRNA | CGCACACAGUGAUGCUUUATT | UAAAGCAUCACUGUGUGCGTT | RNAi |
| *NC*-siRNA | UUCUCCGAACGUGUCACGUTT | ACGUGACACGUUCGGAGAATT |  |

**(C) Validation of the BCC transcriptomes**

| **Primer** | **Sense (5'-3')** | **Antisense (5'-3')** | **Purpose** |
| --- | --- | --- | --- |
| *Tiwaz*-qF | CAAGCTGATCGTGGGCATAC | GTATGCCCACGATCAGCTTG | qRT-PCR |
| *Tiwaz*-qR | GTAGTTGAGGATGTGGCGGA | TCCGCCACATCCTCAACTAC |  |
| *jhamt*-qF | CTCCTTCTACGCCTTGCACT | AGTGCAAGGCGTAGAAGGAG |  |
| *jhamt*-qR | TGTGAGTCGTGGTATGGGGA | TCCCCATACCACGACTCACA |  |
| *RPL38*-qF | GCCCGCAGGAAAGATGCTAA | TTAGCATCTTTCCTGCGGGC |  |
| *RPL38*-qR | GTGAGCATCGCACCTTGAAC | GTTCAAGGTGCGATGCTCAC |  |
| *RBP*-qF | GTTATCGATGCCCCGAGGTT | AACCTCGGGGCATCGATAAC |  |
| *RBP*-qR | CGGAGGAGGAATGGTGTGTC | CGGAGGAGGAATGGTGTGTC |  |
| *CYP18a1*-qF | TGACCATCGTTCTCAGCGAC | GTCGCTGAGAACGATGGTCA |  |
| *CYP18a1*-qR | GCCGAATTCACGCAGTTTGT | ACAAACTGCGTGAATTCGGC |  |
| *chitindeacetylase1*-qF | ACCAAATGCATGCCCCCTTA | TAAGGGGGCATGCATTTGGT |  |
| *chitindeacetylase1*-qR | ACTTCTTAACATTGAAGCCTTCTCC | GGAGAAGGCTTCAATGTTAAGAAGT |  |
| *DDB*-qF | CGGCATAATGCACCAGCAAA | TTTGCTGGTGCATTATGCCG |  |
| *DDB*-qR | CGTAAGACCGAGGGCCATTT | AAATGGCCCTCGGTCTTACG |  |
| *ELOVL7*-qF | ATGGTTCAGTTCGTGCTGGT | ACCAGCACGAACTGAACCAT |  |
| *ELOVL7*-qR | GAAGCCGTTGAGTAGAGGCA | TGCCTCTACTCAACGGCTTC |  |
| *TGM1*-qF | GCTGAACCAGAACAGACCGA | TCGGTCTGTTCTGGTTCAGC |  |
| *TGM1*-qR | TTGCCAGCCTCCATAACCAG | CTGGTTATGGAGGCTGGCAA |  |
| *AKR2E4*-qF | GACCCGGTTCTCACGTCTTT | AAAGACGTGAGAACCGGGTC |  |
| *AKR2E4*-qR | CCAGAATGAAGGCAGGGTGT | ACACCCTGCCTTCATTCTGG |  |
| *DYRK1*-qF | CGTCTCTAGACACAGAACCTACC | GGTAGGTTCTGTGTCTAGAGACG |  |
| *DYRK1*-qR | TTCTGGACCCGCATTGTTCT | AGAACAATGCGGGTCCAGAA |  |
| *scramblase2*-qF | GCAGACGATGGACAAGACGA | TCGTCTTGTCCATCGTCTGC |  |
| *scramblase2*-qR | CATCCAGGTCAGTGGGGAAC | GTTCCCCACTGACCTGGATG |  |

**(D) Validation of the PG transcriptomes**

| **Primer** | **Sense (5'-3')** | **Antisense (5'-3')** | **Purpose** |
| --- | --- | --- | --- |
| *NPC2*-qF | GTGTAGTCCTCTTCACTGCCC | GGGCAGTGAAGAGGACTACAC | qRT-PCR |
| *NPC2*-qR | TGCTGAGACTTGGCACACTT | AAGTGTGCCAAGTCTCAGCA |  |
| *TMLHE*-qf | GCTCTCATCGTGACCTTCGG | GCTCTCATCGTGACCTTCGG |  |
| *TMLHE*-qR | TGGCGTGGTAGCAAGATGAA | TTCATCTTGCTACCACGCCA |  |
| *UGT5*-qF | CACTTGGACCACAGCAGGAA | TTCCTGCTGTGGTCCAAGTG |  |
| *UGT5*-qR | ATCCCCAGCTGAATAGCACG | CGTGCTATTCAGCTGGGGAT |  |
| *HR4*-qF | GCTCCGAATGGAGGAGTACG | GCTCCGAATGGAGGAGTACG |  |
| *HR4*-qR | CGTACCGCTCCTGAATACCC | GGGTATTCAGGAGCGGTACG |  |
| *SNAI2*-qF | GGCAGCTCCTTCACTATCCC | GGGATAGTGAAGGAGCTGCC |  |
| *SNAI2*-qR | GACATTGGTAACGAGGGCGA | TCGCCCTCGTTACCAATGTC |  |
| *pho-scramblase2*-qF | GCAGACGATGGACAAGACGA | TCGTCTTGTCCATCGTCTGC |  |
| *pho-scramblase2*-qR | CATCCAGGTCAGTGGGGAAC | GTTCCCCACTGACCTGGATG |  |
| *FBLN*-qF | CTGCCTCAATGAGCCTGGAA | TTCCAGGCTCATTGAGGCAG |  |
| *FBLN*-qR | GGAGGTGGCTGAGATGGAAC | GTTCCATCTCAGCCACCTCC |  |
| *APOD*-qF | CCGCTACAAGCTGAACAACC | GGTTGTTCAGCTTGTAGCGG |  |
| *APOD*-qR | CGAACTGGTCGTAGTCCGTG | CACGGACTACGACCAGTTCG |  |
| *MYORG*-qF | GCCACGTACCATACTGGCTT | AAGCCAGTATGGTACGTGGC |  |
| *MYORG*-qR | CTGGAGTACCTGACGGCTTG | CAAGCCGTCAGGTACTCCAG |  |
| *NELL*-qF | GGGATATACACGGGACGCTG | CAGCGTCCCGTGTATATCCC |  |
| *NELL*-qR | CATGCGACCTTGCCATGAAC | GTTCATGGCAAGGTCGCATG |  |

**Table S2. Larvae of different stages in the starvation treatment**

1. **L5-48 h**

| **Day** | **0** | **1** | **2** | **3** | **4** | **5** | **6** | **7** | **8** | **9** | **10** |
| --- | --- | --- | --- | --- | --- | --- | --- | --- | --- | --- | --- |
| **Individual** |  |  |  |  |  |  |  |  |  |  |  |
| **1** | 0.1459 | 0.1577 | 0.1463 | 0.1463 | 0.1467 | 0.1392 | 0.1353 | 0.1238 | Died |  |  |
| **2** | 0.1445 | 0.1374 | 0.1272 | 0.1200 | 0.1207 | 0.1201 | 0.1160 | 0.1022 | 0.0918 | Died |  |
| **3** | 0.1322 | 0.1433 | 0.1337 | 0.1265 | 0.1265 | 0.1245 | 0.0987 | 0.0958 | Died |  |  |
| **4** | 0.1325 | 0.1282 | 0.1277 | 0.1284 | 0.1199 | 0.1101 | 0.0980 | 0.0875 | Died |  |  |
| **5** | 0.1654 | 0.1753 | 0.1683 | 0.1571 | 0.1509 | 0.1550 | 0.1309 | 0.1164 | 0.1077 | Died |  |
| **6** | 0.1730 | 0.1695 | 0.1599 | 0.1562 | 0.1405 | 0.1260 | 0.1004 | Died |  |  |  |
| **7** | 0.1637 | 0.1523 | 0.1422 | 0.1482 | 0.1336 | 0.1331 | 0.1331 | 0.1177 | 0.1147 | Died |  |
| **8** | 0.1441 | 0.1517 | 0.1411 | 0.1449 | 0.1279 | 0.1256 | 0.1048 | 0.1007 | Died |  |  |
| **9** | 0.1581 | 0.1596 | 0.1526 | 0.1529 | 0.1379 | 0.1373 | 0.1141 | 0.0962 | Died |  |  |
| **10** | 0.1464 | 0.1395 | 0.1354 | 0.1380 | 0.1187 | 0.1148 | 0.1035 | Died |  |  |  |
| **11** | 0.1517 | 0.1475 | 0.1486 | 0.1304 | 0.1246 | 0.1320 | 0.0996 | 0.0895 | Died |  |  |
| **12** | 0.1215 | 0.1129 | 0.1165 | 0.1103 | 0.1081 | 0.1099 | 0.0930 | 0.0809 | Died |  |  |
| **13** | 0.1523 | 0.1415 | 0.1446 | 0.1392 | 0.1369 | 0.1340 | 0.1274 | 0.1191 | 0.1069 | 0.0982 | Died |
| **14** | 0.1581 | 0.1634 | 0.1621 | 0.1506 | 0.1442 | 0.1427 | 0.1250 | 0.1028 | Died |  |  |
| **15** | 0.1523 | 0.1603 | 0.1486 | 0.1428 | 0.1283 | 0.1240 | 0.0958 | Died |  |  |  |
| **16** | 0.1675 | 0.1688 | 0.1660 | 0.1638 | 0.1659 | 0.1578 | 0.1501 | 0.1382 | 0.1208 | Died |  |
| **Average (g)** | 0.1506 | 0.1506 | 0.1451 | 0.1410 | 0.1332 | 0.1304 | 0.1141 | 0.1054 | 0.1084 |  |  |

1. **L6-0 h**

| **Day** | **0** | **1** | **2** | **3** | **4** | **5** | **6** | **7** | **8** | **9** |
| --- | --- | --- | --- | --- | --- | --- | --- | --- | --- | --- |
| **Individual** |  |  |  |  |  |  |  |  |  |  |
| **1** | 0.1487 | 0.1473 | 0.1378 | 0.1378 | 0.1344 | 0.1284 | 0.1005 | Died |  |  |
| **2** | 0.1391 | 0.1346 | 0.1340 | 0.1359 | 0.1403 | 0.1405 | 0.1370 | 0.1028 | Died |  |
| **3** | 0.1448 | 0.1493 | 0.1494 | 0.1520 | 0.1453 | 0.1413 | 0.1389 | 0.1292 | 0.1140 | Died |
| **4** | 0.1642 | 0.1585 | 0.1458 | 0.1435 | 0.1310 | 0.1248 | 0.0968 | 0.0860 | Died |  |
| **5** | 0.1548 | 0.1562 | 0.1420 | 0.1424 | 0.1268 | 0.0943 | Died |  |  |  |
| **6** | 0.1528 | 0.1536 | 0.1498 | 0.1499 | 0.1272 | 0.1182 | 0.1032 | Died |  |  |
| **7** | 0.1509 | 0.1448 | 0.1448 | 0.1454 | 0.1323 | 0.1298 | 0.1233 | 0.1101 | Died |  |
| **8** | 0.1416 | 0.1468 | 0.1350 | 0.1335 | 0.1262 | 0.1168 | 0.1042 | Died |  |  |
| **9** | 0.1919 | 0.1851 | 0.1812 | 0.1744 | 0.1644 | 0.1316 | 0.1154 | Died |  |  |
| **10** | 0.1653 | 0.1629 | 0.1582 | 0.1527 | 0.1479 | 0.1404 | 0.1357 | 0.1217 | 0.1090 | Died |
| **11** | 0.1830 | 0.1705 | 0.1664 | 0.1666 | 0.1600 | 0.1401 | 0.1268 | Died |  |  |
| **12** | 0.1704 | 0.1690 | 0.1557 | 0.1541 | 0.1436 | 0.1472 | 0.1348 | 0.1098 | Died |  |
| **13** | 0.1433 | 0.1381 | 0.1321 | 0.1309 | 0.1214 | 0.1201 | 0.1097 | Died |  |  |
| **14** | 0.1650 | 0.1562 | 0.1445 | 0.1404 | 0.1312 | 0.1249 | 0.1180 | 0.1079 | Died |  |
| **Average (g)** | 0.1583 | 0.1552 | 0.1483 | 0.1471 | 0.1380 | 0.1285 | 0.1188 | 0.1096 |  |  |

1. **L6-12 h**

| **Day** | **0** | **0.5** | **1** | **2** | **3** | **4** | **5** | **6** | **7** | **8** |
| --- | --- | --- | --- | --- | --- | --- | --- | --- | --- | --- |
| **Individual** |  |  |  |  |  |  |  |  |  |  |
| **1** | 0.2121 | 0.2384 | 0.2268 | 0.2215 | 0.2054 | 0.2050 | 0.1787 | 0.1252 | Pupated |  |
| **2** | 0.1901 | 0.2030 | 0.1981 | 0.1911 | 0.1837 | 0.1745 | 0.1470 | 0.1251 | 0.1127 | Died |
| **3** | 0.1911 | 0.2136 | 0.2036 | 0.2043 | 0.1802 | 0.1720 | 0.1575 | 0.1212 | Pupated |  |
| **4** | 0.2270 | 0.2265 | 0.2252 | 0.2064 | 0.1960 | 0.1600 | 0.1008 | 0.0766 | 0.0743 | Died |
| **5** | 0.2062 | 0.2234 | 0.2106 | 0.2016 | 0.2035 | 0.1804 | 0.0813 | 0.0769 | Died |  |
| **6** | 0.2280 | 0.2385 | 0.2270 | 0.2045 | 0.2093 | 0.1876 | 0.1373 | Died |  |  |
| **7** | 0.2177 | 0.2315 | 0.2165 | 0.2007 | 0.1817 | 0.1733 | 0.1051 | Died |  |  |
| **8** | 0.2093 | 0.2189 | 0.2082 | 0.2052 | 0.1968 | 0.1919 | 0.1609 | 0.1215 | Died |  |
| **9** | 0.2210 | 0.2351 | 0.2668 | 0.2567 | 0.2500 | 0.1597 | 0.1060 | 0.0987 | Pupated |  |
| **10** | 0.2147 | 0.2612 | 0.2446 | 0.2216 | 0.2149 | 0.1919 | 0.0933 | Died |  |  |
| **Average (g)** | 0.2117 | 0.2290 | 0.2227 | 0.2114 | 0.2022 | 0.1796 | 0.1268 | 0.1065 |  |  |

1. **L6-24 h**

| **Day** | **0** | **1** | **2** | **3** | **4** | **5** | **6** |
| --- | --- | --- | --- | --- | --- | --- | --- |
| **Individual** |  |  |  |  |  |  |  |
| **1** | 0.3261 | 0.3674 | 0.3420 | 0.2597 | 0.1485 | 0.1392 | Pupated |
| **2** | 0.3020 | 0.3491 | 0.2968 | 0.1471 | 0.1360 | Pupated |  |
| **3** | 0.2943 | 0.3581 | 0.3655 | 0.2452 | 0.1470 | 0.1373 | Pupated |
| **4** | 0.3045 | 0.3695 | 0.2222 | 0.1413 | 0.1321 | Pupated |  |
| **5** | 0.3133 | 0.3691 | 0.2231 | 0.1415 | 0.1326 | Pupated |  |
| **6** | 0.3244 | 0.3730 | 0.2977 | 0.1508 | 0.1406 | Pupated |  |
| **7** | 0.2947 | 0.3548 | 0.2372 | 0.1488 | 0.1400 | Pupated |  |
| **9** | 0.2821 | 0.3340 | 0.3292 | 0.2519 | 0.1393 | 0.1286 | Pupated |
| **10** | 0.3176 | 0.3444 | 0.3308 | 0.2979 | 0.1447 | 0.1343 | Pupated |
| **11** | 0.3084 | 0.3484 | 0.2814 | 0.1482 | 0.1313 | Pupated |  |
| **12** | 0.3195 | 0.3806 | 0.2851 | 0.1577 | 0.1451 | Pupated |  |
| **13** | 0.3020 | 0.3392 | 0.3359 | 0.2782 | 0.1410 | 0.1317 | Pupated |
| **14** | 0.2893 | 0.3341 | 0.3276 | 0.3173 | 0.1335 | 0.1237 | Pupated |
| **15** | 0.2953 | 0.3473 | 0.3563 | 0.2647 | 0.1375 | 0.1293 | Pupated |
| **16** | 0.2902 | 0.3376 | 0.3301 | 0.2952 | 0.1390 | 0.1271 | Pupated |
| **Average (g)** | 0.3042 | 0.3538 | 0.3041 | 0.2164 | 0.1392 | 0.1314 |  |

1. **L6-48 h**

| **Day** | **0** | **1** | **2** | **3** | **4** | **5** |
| --- | --- | --- | --- | --- | --- | --- |
| **Individual** |  |  |  |  |  |  |
| **1** | 0.4634 | 0.5083 | 0.3458 | 0.2040 | 0.1931 | Pupated |
| **2** | 0.4342 | 0.4453 | 0.2129 | 0.1970 | Pupated |  |
| **3** | 0.4016 | 0.3768 | 0.2192 | 0.2085 | Pupated |  |
| **4** | 0.4309 | 0.3884 | 0.2216 | 0.2097 | Pupated |  |
| **5** | 0.4149 | 0.3865 | 0.2208 | 0.2009 | Pupated |  |
| **6** | 0.4098 | 0.3780 | 0.2155 | 0.1989 | Pupated |  |
| **7** | 0.3938 | 0.3932 | 0.2144 | 0.2002 | Pupated |  |
| **8** | 0.4103 | 0.4269 | 0.2284 | 0.2100 | Pupated |  |
| **9** | 0.4238 | 0.4029 | 0.2232 | 0.2133 | Pupated |  |
| **10** | 0.4149 | 0.4130 | 0.2295 | 0.2122 | Pupated |  |
| **11** | 0.4509 | 0.4155 | 0.2147 | 0.1982 | Pupated |  |
| **12** | 0.4190 | 0.2658 | 0.2475 | 0.2301 | Pupated |  |
| **13** | 0.4093 | 0.2615 | 0.2393 | 0.2295 | Pupated |  |
| **14** | 0.4237 | 0.5539 | 0.2452 | 0.2315 | Pupated |  |
| **15** | 0.4137 | 0.4805 | 0.4224 | 0.2012 | Pupated |  |
| **Average (g)** | 0.4209 | 0.4064 | 0.2467 | 0.2097 |  |  |

1. **CK-48 h**

| **Day** | **0** | **1** | **2** | **3** | **4** |
| --- | --- | --- | --- | --- | --- |
| **Individual** |  |  |  |  |  |
| **1** | 0.3883 | 0.5186 | 0.3026 | 0.2208 | Pupated |
| **2** | 0.4297 | 0.2596 | 0.2349 | 0.2837 | Pupated |
| **3** | 0.4613 | 0.4514 | 0.2547 | 0.2367 | Pupated |
| **4** | 0.4211 | 0.5571 | 0.3112 | 0.2853 | Pupated |
| **5** | 0.4181 | 0.5495 | 0.3016 | 0.2711 | Pupated |
| **6** | 0.3603 | 0.5372 | 0.3572 | 0.2667 | Pupated |
| **7** | 0.3514 | 0.4252 | 0.3218 | 0.2964 | Pupated |
| **8** | 0.3230 | 0.4447 | 0.2995 | 0.2773 | Pupated |
| **9** | 0.3534 | 0.4363 | 0.2516 | 0.2115 | Pupated |
| **10** | 0.4255 | 0.4771 | 0.2597 | 0.2487 | Pupated |
| **11** | 0.4058 | 0.4624 | 0.2867 | 0.2769 | Pupated |
| **12** | 0.3559 | 0.4222 | 0.3157 | 0.2746 | Pupated |
| **Avergae (g)** | 0.4237 | 0.4672 | 0.2810 | 0.2595 |  |

1. **Average weights**

| **Day** | **0** | **0.5** | **1** | **1.5** | **2.5** | **3.5** | **4.5** | **5.5** | **6.5** | **7.5** | **8.5** |
| --- | --- | --- | --- | --- | --- | --- | --- | --- | --- | --- | --- |
| **Groups** |  |  |  |  |  |  |  |  |  |  |  |
| **L5-48 h** | 0.1506 |  | 0.1506 | 0.1451 | 0.1410 | 0.1332 | 0.1304 | 0.1141 | 0.1054 | 0.1084 | Died |
| **L6-0 h** | 0.1506 | 0.1583 |  | 0.1552 | 0.1483 | 0.1471 | 0.1380 | 0.1285 | 0.1188 | 0.1096 | Died |
| **L6-12 h** | 0.1506 | 0.1583 | 0.2117 | 0.2290 | 0.2227 | 0.2114 | 0.2022 | 0.1796 | 0.1268 | 0.1065 | Died or pupated |
| **L6-24 h** | 0.1506 | 0.1583 | 0.2117 | 0.3042 | 0.3538 | 0.3041 | 0.2164 | 0.1392 | 0.1314 | Pupated |  |
| **L6-48 h** | 0.1506 | 0.1583 | 0.2117 | 0.3042 | 0.4209 | 0.4064 | 0.2467 | 0.2097 | Pupated |  |  |
| **CK** | 0.1506 | 0.1583 | 0.2117 | 0.3042 | 0.4237 | 0.4672 | 0.2810 | 0.2595 | Pupated |  |  |

**Table S3. Identified DEGs in the BCC and PG transcriptomes**

| **(A) 20E signaling** | | | | | | | | | | | | | | | |
| --- | --- | --- | --- | --- | --- | --- | --- | --- | --- | --- | --- | --- | --- | --- | --- |
| Gene | L4-24 h-BCC-1 | L4-24 h-BCC-2 | L4-24 h-BCC-3 | L4-24 h-PG-1 | L4-24 h-PG-2 | L4-24 h-PG-3 | L6-24 h-BCC-1 | L6-24 h-BCC-2 | L6-24 h-BCC-3 | L6-24 h-PG-1 | L6-24 h-PG-2 | L6-24 h-PG-3 | Gene_ID | NR_ID | NR_annotation |
| *neverland* | 12.14 | 0.73 | 3.78 | 259.52 | 211.55 | 1224.21 | 7.19 | 9.45 | 3.54 | 471.41 | 354.67 | 85.54 | LOC118281031 | XP_035457380.1 | cholesterol 7-desaturase-like [*Spodoptera frugiperda*] |
| *spook* | 82.14 | 6.91 | 11.26 | 19061.7 | 11458.79 | 19806.33 | 1.67 | 22.78 | 1.89 | 12570.61 | 11030.41 | 5921.6 | LOC118281189 | XP_035457638.1 | cytochrome P450 307a1-like [*Spodoptera frugiperda*] |
| *phantom* | 23.37 | 16.12 | 18.72 | 7452.11 | 10484.12 | 9207.14 | 16.21 | 17.98 | 15.09 | 2725.33 | 2856.61 | 1632.67 | LOC118266714 | KAF9802309.1 | hypothetical protein SFRURICE_008991 [*Spodoptera frugiperda*] |
| *disembodied* | 3.74 | 0.28 | 0.47 | 1090.43 | 971.89 | 1296.6 | 0.31 | 1.1 | 0.32 | 530.72 | 489.82 | 567.52 | LOC118281625 | KAF9798865.1 | hypothetical protein SFRURICE_020429 [*Spodoptera frugiperda*] |
| *shadow* | 0.73 | 0.41 | 0.47 | 372.88 | 285.76 | 732.64 | 0.21 | 0.58 | 0.34 | 206.63 | 163.14 | 91.41 | LOC118269113 | KAF9822649.1 | hypothetical protein SFRURICE_012428 [*Spodoptera frugiperda*] |
| *shade* | 6.6 | 6.98 | 7.97 | 2.81 | 7.71 | 2.07 | 5.89 | 5.77 | 5.74 | 0.82 | 0.62 | 0.6 | LOC118278003 | XP_035452950.1 | ecdysone 20-monooxygenase-like isoform X2 [*Spodoptera frugiperda*] |
| *CYP18A1* | 110.68 | 114.08 | 113.72 | 88.25 | 40.43 | 20.74 | 18.42 | 12.97 | 11.65 | 30.74 | 50.58 | 36.71 | LOC118266961 | XP_035436527.1 | cytochrome P450 18a1-like [*Spodoptera frugiperda*] |
| *PTTH* | 0.08 | 0.12 | 0.12 | 0.08 | 0.12 | 0.12 | 0 | 0 | 0.03 | 0 | 0 | 0.03 | LOC118281630 | XP_035458150.1 | prothoracicotropic hormone-like [*Spodoptera frugiperda*] |
| *torso* | 1.74 | 0.36 | 0.43 | 16.26 | 29.03 | 8.3 | 5.07 | 4.57 | 4.21 | 2.34 | 2.38 | 0.58 | LOC118266498 | KAF9794316.1 | tyrosine-protein kinase receptor torso-like [*Spodoptera frugiperda*] |
| *E74* | 16.63 | 13.12 | 12.55 | 5.26 | 1.05 | 1.86 | 25.19 | 30.09 | 27.3 | 9.42 | 10.14 | 11.93 | LOC118263008 | XP_035430627.1 | ecdysone-induced protein 74-like isoform X4 [*Spodoptera frugiperda*] |
| *E75* | 48.08 | 146.71 | 122.07 | 162.06 | 128.32 | 85.08 | 71.81 | 74.62 | 67.6 | 29.44 | 34.68 | 25.94 | LOC118267232 | KAF9418464.1 | ecdysone-inducible protein E75 isoform X2 [*Spodoptera frugiperda*] |
| *E78C* | 4.41 | 6.96 | 6.97 | 1.52 | 1.43 | 10.37 | 0.76 | 1.25 | 1.18 | 1.51 | 3.1 | 4.35 | LOC118261897 | XP_035428834.1 | ecdysone-induced protein 78C-like isoform X3 [*Spodoptera frugiperda*] |
| *EcR* | 22.46 | 41.94 | 35.34 | 67.2 | 22.41 | 71.26 | 26.81 | 29.79 | 22.33 | 59.11 | 76.26 | 86.92 | LOC118267067 | XP_035436717.1 | ecdysone receptor isoform X5 [*Spodoptera frugiperda*] |
| *USP* | 37.95 | 31.52 | 31.11 | 40.02 | 15.38 | 31.21 | 37.19 | 44.53 | 35.87 | 117.48 | 115.33 | 185.49 | LOC118273518 | AFX60117.1 | nuclear receptor USP isoform 2 [*Spodoptera frugiperda*] |
| *chinmo* | 43.99 | 48.61 | 49.06 | 12.79 | 4.37 | 9.03 | 14.4 | 19.21 | 14.69 | 0.35 | 0.32 | 0.66 | LOC118273024 | XP_035445660.1 | zinc finger protein chinmo-like isoform X1 [*Spodoptera frugiperda*] |
| *BR-C* | 14.54 | 20.16 | 19.6 | 0.8 | 0.12 | 0.32 | 19.7 | 25.22 | 19.13 | 1.76 | 1.74 | 1.21 | LOC118269059 | XP_035439853.1 | broad-complex core protein-like isoform X2 [*Spodoptera frugiperda*] |
| *E93* | 0.42 | 0.61 | 0.54 | 0.72 | 0.06 | 0.24 | 0.5 | 0.68 | 0.43 | 7.54 | 8.08 | 16.6 | LOC118270242 | XP_035441595.1 | mushroom body large-type Kenyon cell-specific protein 1-like isoform X3 [*Spodoptera frugiperda*] |

| **(B) JH signaling** | | | | | | | | | | | | | | | |  |
| --- | --- | --- | --- | --- | --- | --- | --- | --- | --- | --- | --- | --- | --- | --- | --- | --- |
| Gene | L4-24 h-BCC-1 | L4-24 h-BCC-2 | L4-24 h-BCC-3 | L4-24 h-PG-1 | L4-24 h-PG-2 | L4-24 h-PG-3 | L6-24 h-BCC-1 | L6-24 h-BCC-2 | L6-24 h-BCC-3 | L6-24 h-PG-1 | L6-24 h-PG-2 | L6-24 h-PG-3 | Gene_ID | NR_ID | NR_annotation | |
| *jhamt* | 59.27 | 139.42 | 162.41 | 0.02 | 0.05 | 0.48 | 11.94 | 28.23 | 0.76 | 0 | 0 | 0 | LOC126912661 | XP_022814811.1 | juvenile hormone acid O-methyltransferase-like [*Spodoptera litura*] | |
| *Jhe* | 2.65 | 7.27 | 3.28 | 39.39 | 7.06 | 15.27 | 3.47 | 6.28 | 9.02 | 14.53 | 14.53 | 20.08 | LOC118279115 | XP_035454541.1 | uncharacterized protein LOC118279115 [*Spodoptera frugiperda*] | |
| *JHEH* | 154.56 | 374.57 | 370.15 | 122.61 | 413.45 | 418.32 | 346.46 | 279.48 | 269.22 | 118.9 | 167.03 | 317.99 | LOC118279322 | XP_035454881.1 | juvenile hormone epoxide hydrolase-like [*Spodoptera frugiperda*] | |
| *JHDK* | 3.88 | 1.41 | 2.12 | 0.1 | 3.45 | 2.16 | 1.11 | 0.23 | 13.97 | 2.33 | 22.32 | 3.57 | LOC118271854 | XP_035443991.1 | sarcoplasmic calcium-binding proteins I, III, and IV-like [*Spodoptera frugiperda*] | |
| *JHBP* | 39.39 | 25.6 | 34.27 | 125.15 | 189.35 | 38.82 | 72.8 | 1.04 | 90.66 | 1.32 | 21.29 | 1.92 | LOC118276399 | KAF9808943.1 | juvenile hormone-binding protein-like [*Spodoptera frugiperda*] | |
| *CYP15A1* | 39.07 | 168.39 | 205.6 | 0.03 | 0.18 | 2.91 | 17.54 | 60.64 | 1.88 | 0.08 | 0.09 | 0.05 | LOC118272796 | XP_035445369.1 | farnesoate epoxidase-like [*Spodoptera frugiperda*] | |
| *FOHSDR* | 1.08 | 0.71 | 0.54 | 4.36 | 4.02 | 1.42 | 0.16 | 0 | 1.41 | 0.06 | 0.41 | 0.54 | LOC118274846 | XP_035448978.1 | farnesol dehydrogenase-like [*Spodoptera frugiperda*] | |
| *FPPP* | 10.79 | 10.9 | 11.63 | 41.8 | 30 | 30.01 | 12.52 | 12.98 | 9.8 | 21.63 | 29.48 | 44.42 | LOC118276320 | XP_035450475.1 | phosphoglycolate phosphatase 2-like [*Spodoptera frugiperda*] | |
| *ASTA receptor* | 26.38 | 25.32 | 29.98 | 6.05 | 9.96 | 10.76 | 24.17 | 20.19 | 24.47 | 0.66 | 1.09 | 0.61 | LOC118264343 | XP_022830609.1 | allatostatin-A receptor isoform X2 [*Spodoptera litura*] | |

| **(C) Ligard-receptor activity-BCC** | | | | | | | | | | | | |
| --- | --- | --- | --- | --- | --- | --- | --- | --- | --- | --- | --- | --- |
| Gene | L4-24 h-1 | L4-24 h-1 | L4-24 h-1 | L6-24 h-1 | L6-24 h-2 | L6-24 h-3 | log_2_FC | Pvalue | FDR | Gene_ID | NR_ID | NR_annotation |
| *MTH4* | 2.2 | 2.29 | 2.05 | 4.42 | 6.03 | 4.42 | 1.240297 | 6.91E-07 | 1.47E-05 | LOC118272992 | XP_035446318.1 | G-protein coupled receptor Mth-like [*Spodoptera frugiperda*] |
| *DRD1N* | 1.56 | 2 | 2.08 | 0.8 | 1.05 | 0.95 | -1.0075 | 3.86E-06 | 6.76E-05 | LOC118278288 | XP_022834024.1 | dopamine receptor 2-like [*Spodoptera litura*] |
| *DopEcR* | 11.4 | 14.17 | 13.12 | 4.51 | 7.08 | 6.73 | -1.04876 | 1.51E-05 | 0.000223 | LOC118270494 | XP_035442020.1 | G-protein coupled receptor 52-like isoform X1 [*Spodoptera frugiperda*] |
| *TyrR* | 2.5 | 2.83 | 3.04 | 1.3 | 1.32 | 1.42 | -1.04903 | 8.71E-07 | 1.8E-05 | LOC118270634 | XP_035442493.1 | octopamine receptor 1-like [*Spodoptera frugiperda*] |
| *Octbeta* | 6 | 5.73 | 6.48 | 2.12 | 3.56 | 3.05 | -1.05997 | 2.04E-05 | 0.00029 | LOC118270382 | XP_035441849.1 | octopamine receptor beta-2R [*Spodoptera frugiperda*] |
| *FTZ-F1* | 2.27 | 6.81 | 5.28 | 1.78 | 2.33 | 1.34 | -1.12671 | 0.006639 | 0.035384 | LOC118263099 | XP_035430783.1 | nuclear hormone receptor FTZ-F1 beta-like isoform X2 [*Spodoptera frugiperda*] |
| *Oamb* | 5.14 | 3.98 | 3.36 | 1.64 | 2.08 | 2.14 | -1.13261 | 0.003487 | 0.021188 | LOC118270565 | XP_035442079.1 | octopamine receptor Oamb-like isoform X2 [*Spodoptera frugiperda*] |
| *TACR3* | 1.17 | 1.76 | 1.52 | 0.6 | 0.76 | 0.65 | -1.13571 | 6.4E-05 | 0.000769 | LOC118268412 | XP_035438775.1 | tachykinin-like peptides receptor 86C isoform X1 [*Spodoptera frugiperda*] |
| *Octalpha* | 4.57 | 5.34 | 5.26 | 1.72 | 2.79 | 2.34 | -1.14257 | 8.09E-07 | 1.69E-05 | LOC118270235 | XP_035441582.1 | alpha-2Db adrenergic receptor-like [*Spodoptera frugiperda*] |
| *DRD2* | 5.63 | 5.84 | 5.97 | 2.02 | 2.85 | 2.9 | -1.16151 | 1.69E-08 | 5.29E-07 | LOC118267661 | XP_035437663.1 | dopamine D2-like receptor [*Spodoptera frugiperda*] |
| *CHRM3* | 11.01 | 10.07 | 9.51 | 2.59 | 5.96 | 4.17 | -1.27614 | 0.000142 | 0.001521 | LOC118276145 | XP_022828108.1 | muscarinic acetylcholine receptor DM1 [*Spodoptera litura*] |
| *HTR7* | 7.18 | 10.58 | 8.07 | 1.74 | 3.87 | 2.27 | -1.28918 | 6.49E-05 | 0.000778 | LOC118270339 | XP_035441789.1 | 5-hydroxytryptamine receptor 1-like [*Spodoptera frugiperda*] |
| *MTH3* | 7.06 | 4.8 | 6.55 | 3.73 | 1.73 | 1.99 | -1.31973 | 0.001288 | 0.00949 | LOC118275818 | XP_035449800.1 | probable G-protein coupled receptor Mth-like 3 [*Spodoptera frugiperda*] |
| *NPSIFR2* | 1.59 | 1.65 | 1.94 | 0.66 | 0.67 | 0.71 | -1.33801 | 1.8E-14 | 1.44E-12 | LOC118269799 | XP_035441007.1 | neuropeptide SIFamide receptor-like [*Spodoptera frugiperda*] |
| *TRHR* | 1.27 | 2.12 | 1.85 | 0.5 | 0.7 | 0.58 | -1.47397 | 0.000104 | 0.001171 | LOC118270299 | XP_035441718.1 | thyrotropin-releasing hormone receptor-like isoform X1 [*Spodoptera frugiperda*] |
| *GABBR* | 4.67 | 4.2 | 4.05 | 1.53 | 1.76 | 1.69 | -1.50011 | 1.28E-12 | 8.33E-11 | LOC118265550 | XP_035434372.1 | gamma-aminobutyric acid type B receptor subunit 2-like [*Spodoptera frugiperda*] |
| *OPN3* | 1.66 | 1.57 | 1.71 | 0.48 | 0.65 | 0.54 | -1.56825 | 4.51E-06 | 7.7E-05 | LOC118267848 | XP_035437700.1 | parapinopsin-like [*Spodoptera frugiperda*] |
| *CCKAR* | 2.92 | 2.97 | 2.75 | 0.88 | 0.87 | 1.12 | -1.58273 | 7.91E-11 | 3.89E-09 | LOC118263891 | XP_035431891.1 | gastrin/cholecystokinin type B receptor-like [*Spodoptera frugiperda*] |
| *NMUR1* | 2.2 | 1.14 | 1.2 | 0.35 | 0.39 | 0.79 | -1.60547 | 0.0084 | 0.042448 | LOC118271510 | XP_035443068.1 | neuropeptides capa receptor-like isoform X2 [*Spodoptera frugiperda*] |
| *NPFFR2* | 1.9 | 1.28 | 1.15 | 0.42 | 0.4 | 0.52 | -1.69794 | 0.000155 | 0.001642 | LOC118282236 | XP_035459110.1 | neuropeptide FF receptor 2-like [*Spodoptera frugiperda*] |
| *HR38* | 6.59 | 4.79 | 4.27 | 2.15 | 1.42 | 1.28 | -1.7047 | 6.99E-07 | 1.48E-05 | LOC118273391 | XP_035446229.1 | probable nuclear hormone receptor HR38 isoform X1 [*Spodoptera frugiperda*] |
| *GPR84* | 2.71 | 4.36 | 3.79 | 0.49 | 0.9 | 0.95 | -2.19629 | 2.97E-08 | 8.7E-07 | LOC118267410 | XP_035437275.1 | G-protein coupled receptor moody-like [*Spodoptera frugiperda*] |
| *EIP78C* | 4.41 | 6.96 | 6.97 | 0.76 | 1.25 | 1.18 | -2.71315 | 4.12E-24 | 8.35E-22 | LOC118261897 | XP_035428834.1 | ecdysone-induced protein 78C-like isoform X3 [*Spodoptera frugiperda*] |
| *HR3* | 5.36 | 93.4 | 89.78 | 2.81 | 3.24 | 2.96 | -4.3601 | 9.25E-06 | 0.000143 | LOC118279815 | XP_035455499.1 | probable nuclear hormone receptor HR3 isoform X4 [*Spodoptera frugiperda*] |

| **(D) Hydrolase and peptidase activity-BCC** | | | | | | | | | | | | |
| --- | --- | --- | --- | --- | --- | --- | --- | --- | --- | --- | --- | --- |
| Gene | L4-24 h-1 | L4-24 h-1 | L4-24 h-1 | L6-24 h-1 | L6-24 h-2 | L6-24 h-3 | log_2_FC | Pvalue | FDR | Gene_ID | NR_ID | NR_annotation |
| *brachyurin* | 3.02 | 2.05 | 4.29 | 302.8 | 1.92 | 399 | 6.261977 | 2.42E-05 | 0.000334 | LOC118281920 | XP_035458627.1 | brachyurin-like [*Spodoptera frugiperda*] |
| *ACY1* | 0.34 | 1.41 | 1.37 | 85.02 | 60.51 | 28.07 | 5.810764 | 1.44E-26 | 3.35E-24 | LOC118261808 | XP_035428662.1 | aminoacylase-1-like [*Spodoptera frugiperda*] |
| *MYORG* | 0.57 | 0.4 | 0.37 | 24.95 | 0.59 | 42.08 | 5.65513 | 1.54E-05 | 0.000225 | LOC118274208 | XP_035447534.1 | myogenesis-regulating glycosidase-like [*Spodoptera frugiperda*] |
| *trypsin II* | 7.31 | 0.71 | 0.41 | 106 | 1.35 | 313.1 | 5.563508 | 0.001982 | 0.01357 | LOC118273955 | XP_035447132.1 | trypsin II-P29-like [*Spodoptera frugiperda*] |
| *CFT-1* | 20.56 | 11.58 | 15.76 | 678 | 4.35 | 1118 | 5.223466 | 0.000495 | 0.004402 | LOC118274806 | XP_035448427.1 | trypsin CFT-1-like [*Spodoptera frugiperda*] |
| *myrosinase 1* | 0.36 | 0.37 | 0.18 | 8.48 | 9.09 | 9.99 | 4.655053 | 5.47E-20 | 7.67E-18 | LOC118277675 | XP_035452468.1 | myrosinase 1-like [*Spodoptera frugiperda*] |
| *LPH* | 1.26 | 0.29 | 0.27 | 47.31 | 0.96 | 34.3 | 4.554852 | 1.32E-06 | 2.63E-05 | LOC118270693 | XP_035441530.1 | lactase-phlorizin hydrolase-like [*Spodoptera frugiperda*] |
| *ANPEP* | 3.2 | 1.58 | 1.42 | 49.86 | 0.57 | 80.65 | 4.38449 | 0.002223 | 0.014832 | LOC126912773 | QNG41928.1 | aminopeptidase, partial [*Spodoptera frugiperda*] |
| *CPO* | 2.67 | 0.75 | 1.11 | 29.32 | 0.29 | 65.38 | 4.318478 | 0.005114 | 0.028833 | LOC118275823 | XP_035449466.1 | zinc carboxypeptidase-like [*Spodoptera frugiperda*] |
| *pepP* | 0.33 | 0.15 | 0.14 | 5.54 | 0.07 | 5.48 | 4.120438 | 0.000627 | 0.005323 | LOC118280887 | QLC28938.1 | xaa-pro aminopeptidase [*Spodoptera frugiperda*] |
| *alkaline C* | 21.97 | 4.51 | 10.14 | 282.7 | 1.58 | 344.7 | 4.014672 | 0.007311 | 0.038059 | LOC118274813 | XP_035448381.1 | trypsin, alkaline C-like [*Spodoptera frugiperda*] |
| *GBA* | 0.03 | 3.07 | 4.26 | 25 | 31.71 | 33.09 | 3.797392 | 0.004171 | 0.024417 | LOC118274858 | XP_035448491.1 | lysosomal acid glucosylceramidase-like [*Spodoptera frugiperda*] |
| *ZUFSP* | 0.16 | 0.31 | 0.2 | 2.81 | 4.51 | 1.15 | 3.650563 | 8.68E-09 | 2.91E-07 | - | GBP49552.1 | LINE-1 retrotransposable element ORF2 protein [*Eumeta japonica*] |
| *RDP* | 0 | 2.44 | 4.82 | 13.83 | 13.63 | 22.11 | 2.828116 | 0.008447 | 0.042619 | RTase | GBP32168.1 | Probable RNA-directed DNA polymerase from transposon BS [*Eumeta japonica*] |
| *poxin* | 1.26 | 3.26 | 3.49 | 15.43 | 13.9 | 18.77 | 2.749929 | 2.26E-06 | 4.19E-05 | LOC118274833 | XP_035448453.1 | poxin-like isoform X2 [*Spodoptera frugiperda*] |
| *VCL6* | 8.29 | 7.94 | 7.14 | 93.73 | 34.72 | 27.73 | 2.727953 | 3.94E-09 | 1.41E-07 | LOC118269160 | XP_022813957.1 | venom carboxylesterase-6-like [*Spodoptera litura*] |
| *KLKB1* | 4.99 | 6.45 | 7.62 | 28.68 | 23.22 | 19.21 | 2.66749 | 1.8E-30 | 5.07E-28 | LOC126912300 | XP_035454062.1 | serine protease snake-like [*Spodoptera frugiperda*] |
| *MYO1* | 1.43 | 2.45 | 1.05 | 5.16 | 8.35 | 5.93 | 1.981085 | 1.65E-06 | 3.19E-05 | LOC126911523 | XP_022822475.1 | myrosinase 1-like [*Spodoptera litura*] |
| *PLPP* | 0.84 | 0.72 | 0.61 | 3.57 | 3.02 | 2.03 | 1.974451 | 5.11E-07 | 1.14E-05 | LOC118266466 | XP_035435831.1 | putative phosphatidate phosphatase [*Spodoptera frugiperda*] |
| *ARL3* | 3.7 | 5.25 | 6.43 | 14.82 | 12.92 | 12.38 | 1.375804 | 1.77E-06 | 3.4E-05 | LOC118270572 | XP_035442074.1 | ADP-ribosylation factor-like protein 3 isoform X1 [*Spodoptera frugiperda*] |
| *Sp7* | 24.02 | 20.01 | 14.39 | 51.3 | 43.25 | 58.26 | 1.368789 | 1.9E-06 | 3.62E-05 | LOC118265362 | XP_035434089.1 | serine protease 7-like isoform X2 [*Spodoptera frugiperda*] |
| *SLX1* | 3.2 | 1.64 | 2.93 | 6.94 | 5.51 | 5.79 | 1.214988 | 0.004061 | 0.023924 | LOC118275190 | XP_035448953.1 | structure-specific endonuclease subunit SLX1 homolog [*Spodoptera frugiperda*] |
| *NAGA* | 9.36 | 6.63 | 5.07 | 13.93 | 25.29 | 11.53 | 1.21458 | 0.002915 | 0.018343 | LOC118271092 | XP_035442873.1 | alpha-N-acetylgalactosaminidase-like isoform X2 [*Spodoptera frugiperda*] |
| *CPQ* | 92.23 | 65.9 | 68.6 | 181.3 | 137.7 | 172 | 1.097154 | 4.51E-06 | 7.7E-05 | LOC118263679 | XP_035431902.1 | carboxypeptidase Q-like isoform X1 [*Spodoptera frugiperda*] |
| *PPT* | 24.79 | 15.66 | 19.75 | 48.13 | 38.13 | 38.5 | 1.034708 | 5.72E-05 | 0.000697 | LOC118272674 | XP_035445196.1 | palmitoyl-protein thioesterase 1-like [*Spodoptera frugiperda*] |
| *RNF220* | 1.49 | 1.33 | 1.11 | 2.25 | 2.92 | 2.84 | 1.018652 | 0.000324 | 0.003075 | LOC118280958 | XP_035457270.1 | E3 ubiquitin-protein ligase RNF220-like [*Spodoptera frugiperda*] |
| *CEL* | 7.31 | 10.13 | 13.21 | 19.48 | 14.45 | 27.11 | 1.014003 | 0.001822 | 0.012689 | LOC118276221 | XP_035450352.1 | bile salt-activated lipase-like [*Spodoptera frugiperda*] |

| **(E) Proteasome-PG** | | | | | | | | | | | | |
| --- | --- | --- | --- | --- | --- | --- | --- | --- | --- | --- | --- | --- |
| Gene | L4-24 h-1 | L4-24 h-2 | L4-24 h-3 | L6-24 h-1 | L6-24 h-2 | L6-24 h-3 | log_2_FC | Pvalue | FDR | Gene_ID | NR_ID | NR_annotation |
| *SIAH1* | 0.08 | 0.63 | 1.11 | 0.68 | 10.97 | 13.12 | 3.260292 | 0.00296 | 0.024249 | LOC118272611 | XP_035445111.1 | putative E3 ubiquitin-protein ligase SINAT1 isoform X1 [*Spodoptera frugiperda*] |
| *RNF34* | 5.27 | 2.13 | 5.92 | 10.02 | 10.45 | 11.47 | 1.031913 | 0.00274 | 0.022843 | LOC118267334 | XP_035437162.1 | E3 ubiquitin-protein ligase rififylin-like [*Spodoptera frugiperda*] |
| *USP3* | 27.33 | 11.27 | 13.63 | 9.56 | 7.57 | 12.18 | -1.11374 | 0.003881 | 0.030289 | LOC118273967 | XP_035447149.1 | ubiquitin carboxyl-terminal hydrolase 3-like [*Spodoptera frugiperda*] |
| *RNF5* | 77.71 | 28.92 | 55.09 | 26.23 | 28.95 | 29.66 | -1.16352 | 0.000147 | 0.001909 | LOC118267034 | CAB3234786.1 | Predicted E3 ubiquitin ligase [*Arctia plantaginis*] |
| *KCMF1* | 76.73 | 64.95 | 53.04 | 35.38 | 37.19 | 39.05 | -1.20503 | 0.005403 | 0.039841 | LOC118277852 | XP_035452712.1 | E3 ubiquitin-protein ligase KCMF1-like isoform X1 [*Spodoptera frugiperda*] |
| *UBE3C* | 22.97 | 10.67 | 17.07 | 7.7 | 7.14 | 8.73 | -1.38099 | 1.61E-06 | 3.7E-05 | LOC118269654 | XP_035440762.1 | ubiquitin-protein ligase E3C-like [*Spodoptera frugiperda*] |
| *UBC* | 894.4 | 558.4 | 1192 | 364.5 | 397.5 | 429 | -1.45309 | 2.01E-05 | 0.000343 | LOC118274278 | XP_038013558.1 | polyubiquitin-C isoform X1 [*Spodoptera frugiperda*] |
| *ARIH1* | 53.86 | 24.55 | 20.01 | 13.43 | 14.1 | 16.03 | -1.47571 | 0.001017 | 0.009928 | LOC118273952 | XP_035447125.1 | E3 ubiquitin-protein ligase ariadne-1-like isoform X1 [*Spodoptera frugiperda*] |
| *NGLY1* | 24.94 | 15.81 | 17.92 | 7.24 | 7.27 | 10.52 | -1.58395 | 1.07E-05 | 0.000196 | LOC118266530 | XP_035435900.1 | peptide-N(4)-(N-acetyl-beta-glucosaminyl) asparagine amidase-like [*Spodoptera frugiperda*] |
| *UBE4A* | 34.1 | 14.41 | 25.65 | 7.9 | 9.29 | 10.29 | -1.69091 | 9.16E-09 | 3.55E-07 | LOC118268724 | XP_035439225.1 | ubiquitin conjugation factor E4 A-like [*Spodoptera frugiperda*] |
| *USP20* | 36.88 | 24.38 | 44.11 | 10.84 | 12.87 | 16 | -1.72952 | 1.76E-07 | 5.12E-06 | LOC118277299 | XP_035451914.1 | ubiquitin carboxyl-terminal hydrolase 20-like [*Spodoptera frugiperda*] |
| *PSMA5* | 474.8 | 514.2 | 508.3 | 192.6 | 188.3 | 187.9 | -1.82534 | 4.51E-05 | 0.000686 | LOC118279178 | XP_021199618.1 | proteasome subunit alpha type-5 [*Helicoverpa armigera*] |
| *RAD23* | 425.4 | 431.5 | 337.5 | 155.5 | 143.8 | 146 | -1.85097 | 7.2E-05 | 0.001029 | LOC118267122 | XP_035436814.1 | UV excision repair protein RAD23 homolog A-like [*Spodoptera frugiperda*] |
| *PSMD11* | 720.9 | 551.6 | 669.4 | 220.8 | 220.3 | 228 | -1.89214 | 8.87E-08 | 2.79E-06 | LOC118269382 | XP_035440341.1 | 26S proteasome non-ATPase regulatory subunit 11-like [*Spodoptera frugiperda*] |
| *NPLOC4* | 45.88 | 52.55 | 42.4 | 14.92 | 14.78 | 18.71 | -1.99792 | 2.58E-05 | 0.000425 | LOC118264773 | XP_035433294.1 | nuclear protein localization protein 4 homolog [*Spodoptera frugiperda*] |
| *RBX1* | 878.8 | 1838 | 702.6 | 471.2 | 450.5 | 331.2 | -2.00718 | 0.003533 | 0.028035 | LOC118263323 | NP_001040275.1 | E3 ubiquitin-protein ligase ring box protein [*Bombyx mori*] |
| *OTU1* | 67.11 | 29.7 | 37.49 | 13.18 | 13.58 | 10.83 | -2.09682 | 4.61E-08 | 1.53E-06 | LOC118268297 | XP_035438627.1 | ubiquitin thioesterase OTU1-like [*Spodoptera frugiperda*] |
| *RNF181* | 68.88 | 57.31 | 59.94 | 20.16 | 18.51 | 15.63 | -2.14315 | 5.94E-07 | 1.53E-05 | LOC118269620 | XP_035440700.1 | E3 ubiquitin-protein ligase RNF181-like [*Spodoptera frugiperda*] |
| *PSMA1* | 334.9 | 358 | 309.9 | 86.26 | 94.82 | 111.2 | -2.2188 | 8.92E-07 | 2.18E-05 | LOC118271282 | XP_035443196.1 | proteasome subunit alpha type-1-like isoform X3 [*Spodoptera frugiperda*] |
| *PSMD1* | 525.9 | 367.6 | 500.8 | 107.8 | 123.1 | 132.7 | -2.27952 | 2.29E-12 | 1.89E-10 | LOC118267453 | XP_035437352.1 | 26S proteasome non-ATPase regulatory subunit 1-like [*Spodoptera frugiperda*] |
| *USP22* | 35.37 | 11.09 | 6.39 | 4.23 | 3.76 | 4.16 | -2.35322 | 0.000104 | 0.00142 | LOC118264077 | XP_035432319.1 | ubiquitin carboxyl-terminal hydrolase nonstop-like [*Spodoptera frugiperda*] |
| *PSMD7* | 576.2 | 778.3 | 538.7 | 180.8 | 168.7 | 163.3 | -2.3622 | 7.81E-06 | 0.00015 | LOC118273461 | XP_035445748.1 | 26S proteasome non-ATPase regulatory subunit 7-like [*Spodoptera frugiperda*] |
| *PSMA4* | 693.1 | 1302 | 766.3 | 259 | 265.6 | 253.9 | -2.36615 | 8.12E-05 | 0.001145 | LOC126910828 | XP_022817177.1 | proteasome subunit alpha type-4 [*Spodoptera litura*] |
| *PSMA7* | 1067 | 1836 | 1108 | 387.3 | 383.8 | 336.8 | -2.37589 | 5.46E-05 | 0.000811 | LOC118273101 | XP_035445773.1 | proteasome subunit alpha type-7-1-like [*Spodoptera frugiperda*] |
| *PSMB3* | 665.4 | 1315 | 735.1 | 251.7 | 260.6 | 236.7 | -2.40632 | 9.77E-05 | 0.001345 | LOC118274000 | XP_022824678.1 | proteasome subunit beta type-3 [*Spodoptera litura*] |
| *PSMA3* | 676.6 | 1247 | 742.3 | 259.1 | 247.7 | 213.3 | -2.41661 | 6.34E-05 | 0.000928 | LOC118267530 | XP_035437468.1 | proteasome subunit alpha type-3-like [*Spodoptera frugiperda*] |
| *PSMA2* | 794.9 | 1564 | 832.8 | 267 | 282 | 305.2 | -2.46456 | 6.47E-05 | 0.000944 | LOC118278603 | XP_030033604.1 | proteasome subunit alpha type-2 [*Spodoptera litura*] |
| *PSMC4* | 706.1 | 728.3 | 547.5 | 138.5 | 151.2 | 181.4 | -2.52017 | 7.47E-08 | 2.4E-06 | LOC118281902 | XP_022826943.1 | 26S proteasome regulatory subunit 6B [*Spodoptera litura*] |
| *PSMC5* | 816.6 | 1258 | 779.8 | 236.1 | 223.7 | 238.8 | -2.54327 | 5.26E-06 | 0.000105 | LOC118277121 | XP_021199587.1 | 26S protease regulatory subunit 8 [*Helicoverpa armigera*] |
| *PSMB2* | 1032 | 1902 | 1134 | 333.9 | 349.8 | 297.9 | -2.57992 | 1.83E-05 | 0.000316 | LOC118281880 | XP_035458541.1 | proteasome subunit beta type-2-like [*Spodoptera frugiperda*] |
| *PSMB6* | 618.5 | 1088 | 601.7 | 182.4 | 168.7 | 196.5 | -2.62064 | 1.06E-05 | 0.000194 | LOC118263013 | XP_035430638.1 | proteasome subunit beta type-6-like [*Spodoptera frugiperda*] |
| *PSMD6* | 480.8 | 856.8 | 443.5 | 127.5 | 136.1 | 152.3 | -2.65446 | 1.13E-05 | 0.000204 | LOC118273557 | XP_035446488.1 | 26S proteasome non-ATPase regulatory subunit 6-like [*Spodoptera frugiperda*] |
| *PSMD4* | 641.5 | 812.7 | 492.7 | 145.8 | 141.5 | 141.4 | -2.6661 | 6.5E-07 | 1.66E-05 | LOC118281767 | XP_035458375.1 | 26S proteasome non-ATPase regulatory subunit 4-like [*Spodoptera frugiperda*] |
| *PSMB7* | 687.6 | 1360 | 703.6 | 208.1 | 214 | 203.1 | -2.69653 | 1.6E-05 | 0.000277 | LOC118273178 | XP_035445899.1 | proteasome subunit beta type-7-like [*Spodoptera frugiperda*] |
| *PSMD3* | 517.9 | 481.4 | 437.5 | 89.86 | 103 | 95.96 | -2.71813 | 2.62E-10 | 1.43E-08 | LOC118273449 | XP_022820935.1 | probable 26S proteasome non-ATPase regulatory subunit 3 [*Spodoptera litura*] |
| *PSMB5* | 805.6 | 1687 | 792.5 | 216.4 | 226.1 | 230.9 | -2.86732 | 8.47E-06 | 0.000161 | LOC118277502 | XP_022818673.1 | proteasome subunit beta type-5 [*Spodoptera litura*] |
| *PSMC3* | 764 | 980.7 | 608 | 144.7 | 146.7 | 153.9 | -2.88868 | 5.23E-08 | 1.72E-06 | LOC118275405 | XP_021194512.1 | 26S protease regulatory subunit 6A-B [*Helicoverpa armigera*] |
| *PSMB4* | 609.4 | 1237 | 606.4 | 143.8 | 174.3 | 162.3 | -2.92281 | 4.43E-06 | 9.09E-05 | LOC118279861 | XP_035455587.1 | proteasome subunit beta type-4-like [*Spodoptera frugiperda*] |
| *RPN13* | 316.9 | 374.3 | 302 | 50.58 | 59.01 | 67.28 | -2.95418 | 7.68E-10 | 3.78E-08 | LOC118278435 | XP_035453526.1 | proteasomal ubiquitin receptor ADRM1-like isoform X3 [*Spodoptera frugiperda*] |
| *PSMC2* | 794.7 | 1183 | 743.8 | 154 | 156.7 | 181.2 | -2.9816 | 5.44E-08 | 1.78E-06 | LOC118271650 | XP_035443661.1 | 26S proteasome regulatory subunit 7 [*Spodoptera frugiperda*] |
| *PSMC6* | 725 | 1144 | 541.4 | 131.8 | 114.3 | 145 | -3.17065 | 1.38E-07 | 4.14E-06 | LOC118270701 | XP_022827611.1 | 26S proteasome regulatory subunit 10B [*Spodoptera litura*] |
| *PSMD13* | 638.4 | 1187 | 549.5 | 108 | 102.7 | 102.5 | -3.48529 | 3.19E-08 | 1.11E-06 | LOC118280467 | XP_035456404.1 | 26S proteasome non-ATPase regulatory subunit 13-like [*Spodoptera frugiperda*] |
